# Comparison of long-term outcomes of endoscopic submucosal dissection and surgery for undifferentiated-type early gastric cancer meeting the expanded criteria: a systematic review and meta-analysis

**DOI:** 10.1007/s00464-022-09126-9

**Published:** 2022-02-22

**Authors:** Hyo-Joon Yang, Jie-Hyun Kim, Na Won Kim, Il Ju Choi

**Affiliations:** 1 Division of Gastroenterology, Department of Internal Medicine and Gastrointestinal Cancer Center, Kangbuk Samsung Hospital, Sungkyunkwan University School of Medicine, Seoul, Republic of Korea; 2 Division of Gastroenterology, Department of Internal Medicine, Gangnam Severance Hospital, Yonsei University College of Medicine, Seoul, Republic of Korea; 3 Yonsei University Medical Library, Yonsei University, Seoul, Korea; 4 Center for Gastric Cancer, National Cancer Center, 323 Ilsan-ro, Ilsandong-gu, Goyang, Gyeonggi 10408 Republic of Korea

**Keywords:** Endoscopic submucosal dissection, Surgery, Undifferentiated histology, Stomach neoplasms, Systematic review

## Abstract

**Background:**

There have been concerns over the long-term outcomes of endoscopic submucosal dissection (ESD) for undifferentiated-type early gastric cancer (UD EGC). We aimed to compare the long-term outcomes of ESD and surgery for patients with UD EGC.

**Methods:**

We searched PubMed, Embase, and Cochrane Library databases through March 2021 to identify studies that compared the long-term outcomes of ESD and surgery for UD EGC meeting expanded criteria for curative resection. The risk of bias was assessed with the Cochrane tool for non-randomized studies. The risk ratio (RR) was estimated using a fixed-effect model.

**Results:**

Overall, 1863 patients from five retrospective cohort studies, including 908 patients with propensity score matching (PSM), were eligible for meta-analysis. ESD was associated with inferior overall survival (OS) compared to surgery in the overall cohort (RR 2.11; 95% CI 1.26–3.55) but not in the PSM cohort (RR 1.18; 95% CI 0.60–2.32). In the PSM cohort, ESD had a lower disease-free survival (DFS) (RR 2.49; 95% CI 1.42–4.35) and higher recurrence (RR 12.61; 95% CI 3.43–46.37), gastric recurrence (RR 11.25; 95% CI 3.06–41.40), and extragastric recurrence (RR 4.23; 95% CI 0.47–37.93). Recurrence outcomes were similar between the overall and PSM cohorts. Disease-specific survival was not significantly different between the two groups in both the overall and PSM cohorts.

**Conclusion:**

Although OS after curative ESD for UD EGC was not different from that after surgery in the PSM cohort, DFS and recurrence were inferior after ESD. Limitations included a lack of randomized trials. Further prospective studies comparing the long-term outcomes of ESD and surgery for UD EGC are needed (PROSPERO CRD 42021237097).

**Supplementary Information:**

The online version contains supplementary material available at 10.1007/s00464-022-09126-9.

Endoscopic submucosal dissection (ESD) is indicated for early gastric cancer (EGC) with a very low risk of lymph node (LN) metastasis [1–3]. Differentiated-type EGC that were previously considered as an expanded indication, or intramucosal cancer > 3 cm in size without ulceration or ≤ 3 cm in size with ulceration, has been changed to an absolute indication in the latest version of the Japanese Gastric Cancer Association (JGCA) and Japanese Gastroenterological Endoscopy Society (JGES) guidelines [1, 2]. Recently, a Japanese multicenter prospective study reported that no LN recurrence, distant recurrence, or gastric cancer death occurred during 5 years after curative ESD among 195 patients with undifferentiated-type (UD) EGC meeting the expanded criteria defined as intramucosal cancer ≤ 2 cm in size without ulceration [4]. Accordingly, UD EGC meeting the expanded indication was also integrated into the absolute indication category in the JGES guidelines [1]. However, it is still considered an expanded indication in the JGCA and Korean guidelines [2, 3].

There have been concerns over the long-term outcomes of ESD for UD EGC that met the expanded criteria. In an analysis of a prospective surgical database, 310 UD EGCs showed no LN metastasis [5]. However, several other surgical studies reported cases of LN metastasis in UD EGC that met the same criteria [6–9]. Several case reports also described UD EGCs that satisfied the expanded criteria but had LN metastasis [10–12]. Moreover, previous retrospective studies have reported cases of extragastric recurrence years after curative ESD for UD EGC [13, 14]. In addition, poorly differentiated adenocarcinoma (PDA) was reported to harbor a higher risk of LN metastasis than signet ring cell carcinoma (SRC) [15].

In recent years, several systematic reviews reported that overall survival (OS) was comparable between ESD and surgery for patients with EGC meeting the expanded criteria [16–20]. However, these studies included both differentiated-type and UD EGC and did not evaluate the outcomes for UD EGC separately. Because differentiated-type EGCs are dominant among the EGCs meeting the expanded indication, it may be difficult to apply these results to UD EGC. Although one systematic review compared ESD and surgery specifically for UD EGC, this study was also limited because it included some duplicated data and lacked meta-analysis of propensity score matching (PSM) analyses, resulting in possible biased results in the survival and recurrence outcomes [21]. In addition, several new eligible studies have been published.

Therefore, we performed a systematic review and meta-analysis to compare the long-term outcomes of ESD and surgery for patients with UD EGC that met the expanded criteria for curative resection. We included a meta-analysis of PSM analyses and subgroup analyses of PDA and SRC.

## Methods

### Search strategy and study selection

We conducted a systematic literature search using the PubMed, Embase, and Cochrane Library databases from their inception date to March 23, 2021. The search terms included “early gastric cancer,” “early gastric neoplasm,” “early stomach cancer,” “early stomach neoplasm,” “endoscopic submucosal dissection,” “endoscopic mucosal resection,” “endoscopic resection,” “gastrectomy,” and “surgery.” There were no language restrictions. An experienced medical librarian designed and performed a detailed search strategy based on the input from study investigators (Supplemental Table 1). The reference lists of relevant reviews and retrieved articles were manually searched. Two investigators (H.J.Y. and J.H.K.) independently screened all titles and abstracts, examined the full texts of selected studies for eligibility, and resolved discrepancies by consensus. We included randomized controlled trials (RCTs) and prospective and retrospective cohort studies that met the following criteria: (1) patients with UD EGC who underwent curative ESD or surgery were included as the main study population or as a subgroup population; (2) ESD was compared to surgery, including subtotal and total gastrectomy; and (3) either OS, disease-free survival (DFS), or disease-specific survival (DSS) were reported. We excluded (1) studies that excluded UD histology among EGC; (2) studies that included UD EGC but did not specify UD EGC as a group or subgroup; (3) studies with no data on the inclusion of UD EGC; (4) studies that included patients with a previous history of gastric cancer or gastroesophageal surgery; (5) studies that did not report any long-term outcomes; and (6) studies with duplicated data.

The study protocol was exempted from ethics approval by the Institutional Review Board of the National Cancer Center, Korea (IRB No. NCC 2021-0081) and registered at the International Prospective Register of Systematic Reviews (PROSPERO, registration number: CRD 42021237097). We reported this systematic review according to the Preferred Reporting Items for Systematic Reviews and Meta-analysis (PRISMA) 2020 statement [22].

### Data extraction and risk of bias assessment

Two investigators (H.J.Y. and J.H.K.) independently extracted the data using a Microsoft Excel spreadsheet (Microsoft, Redmond, WA, USA). We collected data on study design, country of origin, study period, the number of patients, mean age, the proportion of male patients, and follow-up duration. Outcome data were extracted on number and cause of death and number and type of recurrence both in the overall cohort and in the PSM cohort, if available. Any disagreements between the two investigators were resolved by consensus. We contacted the first or corresponding authors of the included studies to request missing or unreported data. We also contacted the authors to obtain the most up-to-date data when multiple articles contained duplicated data.

Two investigators (H.J.Y. and J.H.K.) independently assessed the risk of bias using the Risk of Bias In Non-randomized Studies of Interventions (ROBINS-I) [23], which was visualized using a visualization tool [24]. If there were any discrepancies, a consensus was reached by discussion between the two investigators. In this study, a single risk of bias assessment was performed across all outcomes because the outcomes were similar in terms of follow-up and ascertainment of outcome events.

### Definitions and outcomes

UD EGC was defined as PDA, SRC, or mucinous adenocarcinoma [2, 25]. Curative ESD was defined as en bloc resection, negative horizontal margin, negative vertical margin, tumor size ≤ 2 cm, intramucosal cancer, absence of ulceration, and absence of lymphovascular invasion [2].

The main outcomes were OS, DFS, and DSS. OS was defined as the time from the date of diagnosis to the date of death from any cause. DFS was defined as the time from the initial treatment to the date of recurrence or death from any cause. DSS was defined as the time from the date of diagnosis to the date of death from gastric cancer. Recurrence was defined as local, metachronous, regional LN, or distant recurrence, while synchronous recurrence was not considered a recurrence. Metachronous and synchronous recurrences were defined as cancer diagnosed at previously uninvolved sites after and within 12 months of initial treatment, respectively. Local recurrence was defined as cancer recurrence at the ESD or the surgical anastomosis site. Local and metachronous recurrences were classified as gastric recurrences, and regional LN and distant recurrences were classified as extragastric recurrences.

### Data synthesis and statistical analysis

The risk ratio (RR) with 95% confidence interval (CI) was calculated using the Mantel–Haenszel fixed-effect model to compare outcomes between ESD and surgery. This was because RR was available in all included studies, while hazard ratio (HR) was available in only two studies. The meta-analysis results were presented using forest plots. Study heterogeneity was assessed using Cochrane’s *Q* test and the *I*^2^ statistics. Where *P* < 0.10 for *Q* test or *I*^2^ > 50%, the random effects model was used. We conducted a pre-specified subgroup analysis to compare outcomes between the two main pathological subtypes of UD histology: PDA and SRC. A post hoc sensitivity analysis was conducted using HR instead of RR as an effect measure. In studies where HR was not available, it was estimated using a method suggested by Tierney et al. [26]. We planned to evaluate publication bias for any comparison made with ≥ 10 studies. However, post hoc funnel plots were generated for all meta-analyses although they included four or five studies. The certainty in the synthesized outcomes was assessed independently by two investigators (H.J.Y. and J.H.K.) using GRADEpro GDT software to generate a summary of findings tables [27]. The statistical analysis was performed using Review Manager (RevMan, version 5.3, Cochrane Collaboration, Copenhagen, Denmark).

## Results

### Study selection and characteristics

**Fig. 1:**
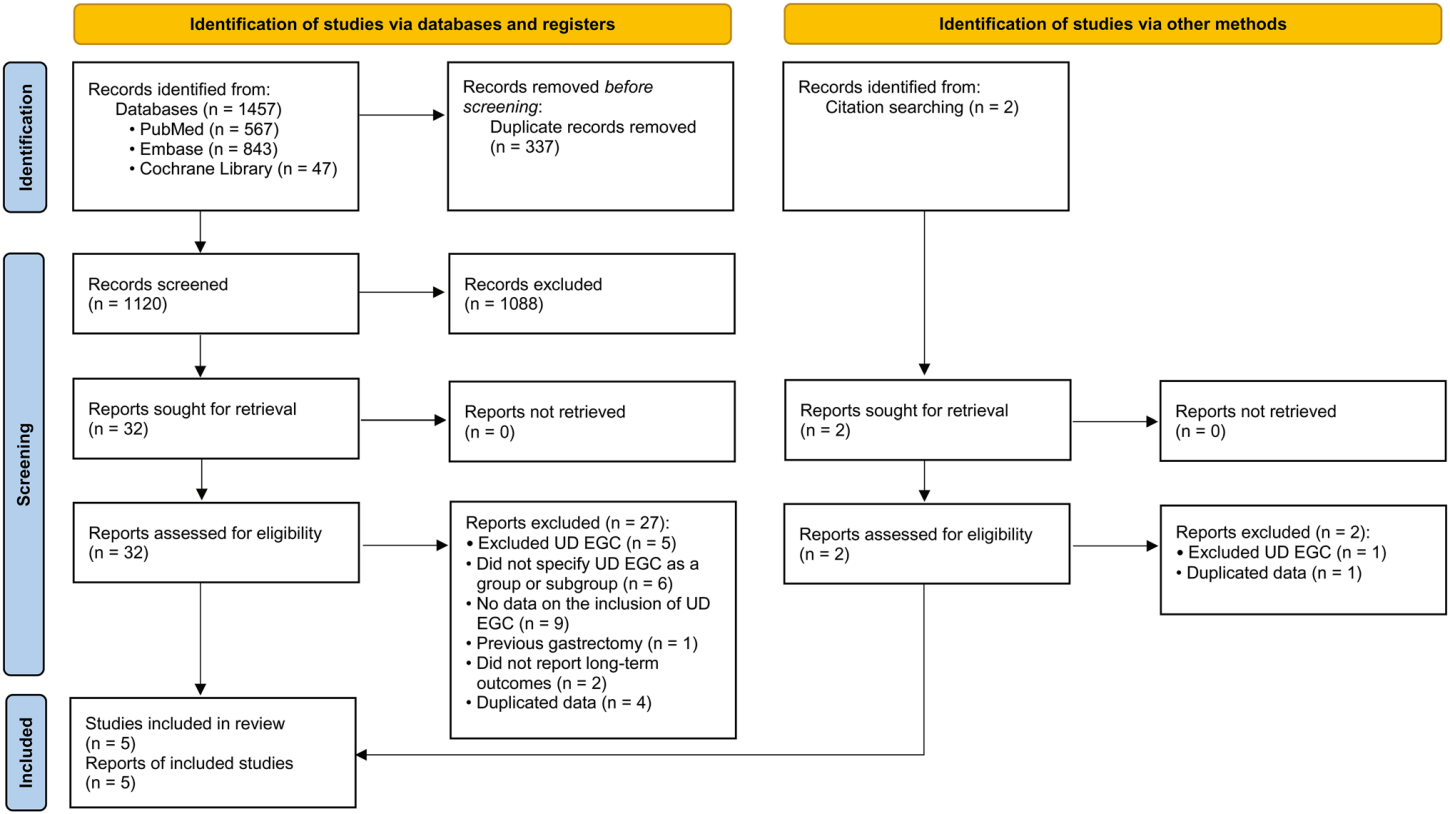
Flow chart of study enrollment. *UD EGC* undifferentiated-type early gastric cancer

The search strategy returned 1479 results, which were narrowed to 34 articles after the removal of duplicates and review of study titles and abstracts (Fig. 1). During the full-text review, 29 articles were excluded. The characteristics of the excluded studies are listed in Supplemental Table 2. Based on these, five articles that fulfilled the eligibility criteria were included [13, 14, 28–30]. None of the studies were RCTs; they were all retrospective cohort studies.

The five studies from Korea and China included 2050 patients with UD EGC from 2005 to 2017 (Table 1). The mean age was significantly higher in the ESD group than in the surgery group in four of the five studies with a median difference of 5.2 years (range 3.6–7.5 years). Furthermore, patients in the ESD group had more underlying illnesses than those in the surgery group in three studies. The median follow-up duration ranged from 47.1 to 75.6 months, and the survival and recurrence outcomes were retrieved from all five studies. Four studies conducted PSM analysis. Because two studies included patients who underwent noncurative ESD for UD EGC, we contacted the authors and provided data for patients with curative ESD only [13, 30]. In addition, there were some data duplications between the two studies [14, 29]. Therefore, we contacted the authors and provided data after excluding duplicated records. Finally, we included 1863 patients with UD EGC meeting the expanded criteria who underwent curative ESD (*n* = 549) or surgery (*n* = 1314) for meta-analysis of overall cohort and 908 patients who underwent ESD (*n* = 400) or surgery (*n* = 508) for meta-analysis of PSM cohort. All studies were included in all outcome syntheses.

**Table 1:** Characteristics of studies included in meta-analyses

Author (year) [ref]	Country	Study design	Study period	Patients, *n*		Mean age, years		Male sex, %		Pathology specimen cutting interval, mm		Curative resection, %	Follow-up, months		PSM
				ESD	Surgery	ESD	Surgery	ESD	Surgery	ESD	Surgery		ESD	Surgery	
Ahn et al. (2021) [14]	Korea	Retrospective cohort study	2005–2015	328	383	58.4	50.9	52.1	45.2	2	4–6	100	75.6	75.6	Yes
Guo et al. (2020) [28]	China	Retrospective cohort study	2010–2017	40	52	61.8	58.2	65.0	59.6	2	5	100	70.0	76.0	No
Lee et al. (2018) [30]	Korea	Retrospective cohort study	2005–2013	83	341	64.0	57.5	73.0	61.8	2	4 – 5	95.7	50.2	59.5	Yes
Lim et al. (2019) [29]	Korea	Retrospective cohort study	2007–2014	48	282	59.9	54.7	52.4	47.4	2	4	100	65.9	58.3	Yes
Park et al. (2018) [13]	Korea	Retrospective cohort study	2006–2012	111	382	57.3	52.8	54.1	44.0	2	4	88.3	47.1	60.2	Yes

*PSM* propensity score matching, *ESD* endoscopic submucosal dissection

The risk of bias assessment of all included studies is provided in Supplemental Fig. 1. Although these were retrospective cohort studies, we scored all the studies with low risk of bias associated with selection of participants, classification of interventions, deviations of interventions, and outcome measurement. There were several studies with moderate risk of bias due to confounding, missing data, or selective reporting, and one study had a serious risk of bias due to confounding.

### Overall survival

**Fig. 2:**
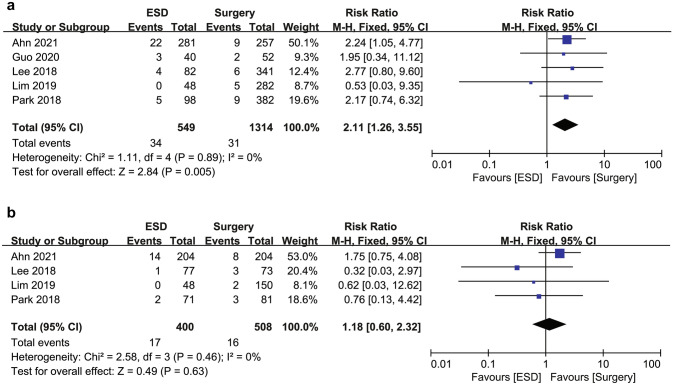
Forest plot of overall survival for endoscopic submucosal dissection and surgery for undifferentiated-type early gastric cancer (**a**) in the overall cohort and (**b**) in the propensity score matching cohort. *ESD* endoscopic submucosal dissection

The OS rate was significantly lower in the ESD group (515/549, 94.2%) than in the surgery group (1283/1314, 97.6%), with an RR for mortality of 2.11 (95% CI 1.26–3.55; *I*^2^ = 0%) in the overall cohort (Fig. 2). However, in the PSM cohort, OS rates were not significantly different between the ESD (383/400, 95.8%) and surgery (492/508, 96.9%) groups with a decreased RR of 1.18 (95% CI 0.60–2.32; *I*^2^ = 0%). These results suggest that the increased RR for mortality associated with the ESD group in the overall cohort might be attributable to the potential selection bias of the individual studies. No statistical heterogeneity was detected in the analysis of OS in both the overall and PSM cohorts.

### Disease-free survival and recurrence

**Fig. 3:**
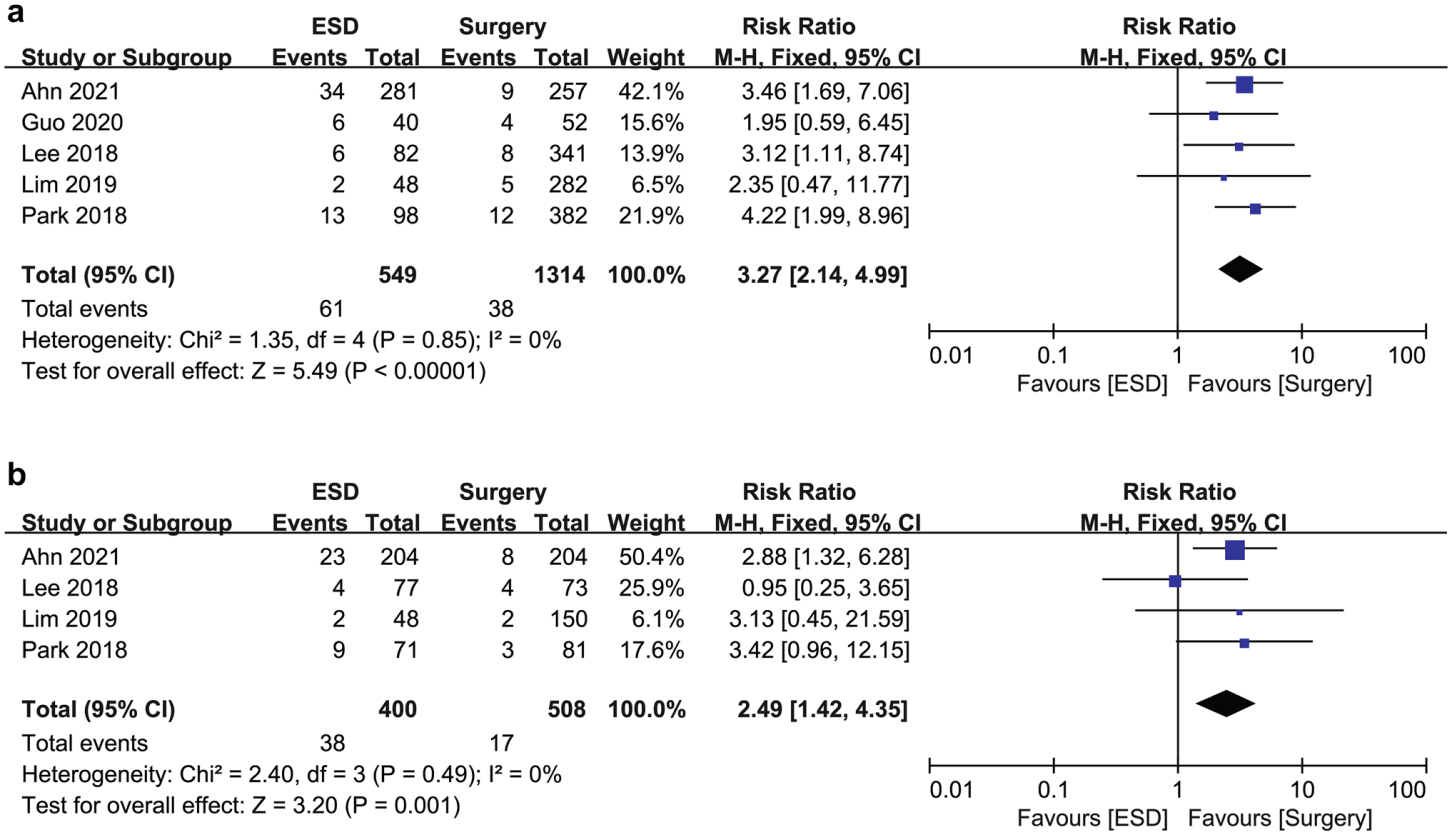
Forest plot of disease-free survival for endoscopic submucosal dissection and surgery for undifferentiated-type early gastric cancer (**a**) in the overall cohort and (**b**) in the propensity score matching cohort. *ESD* endoscopic submucosal dissection

DFS rate was significantly lower in the ESD group than in the surgery group both in the overall cohort (88.9% [488/549] vs. 97.1% [1276/1314]; RR 3.27; 95% CI 2.14–4.99; *I*^2^ = 0%) and in the PSM cohort (90.5% [362/400] vs. 96.7% [491/508]; RR 2.49; 95% CI 1.42–4.35; *I*^2^ = 0%) (Fig. 3).

**Fig. 4:**
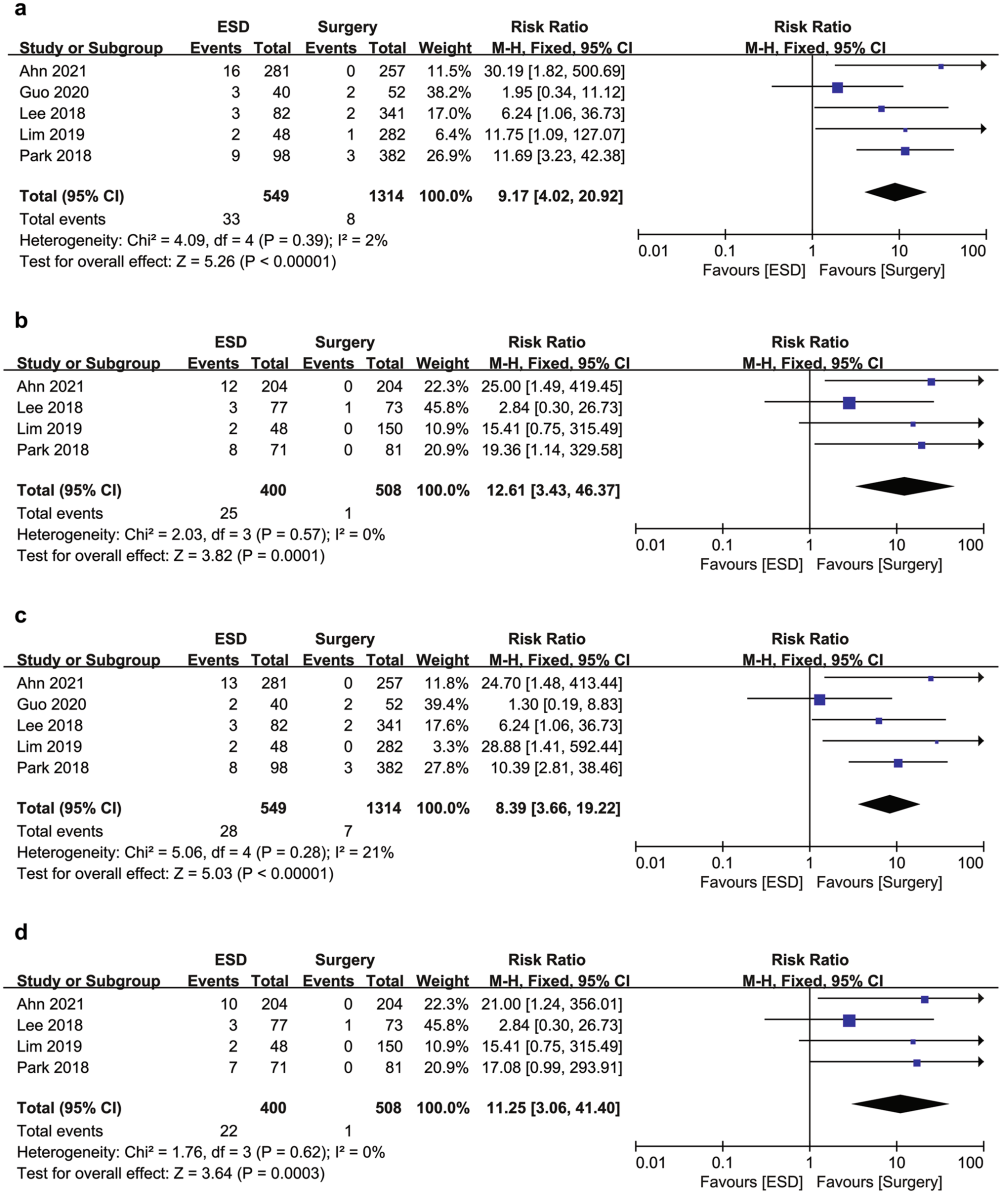

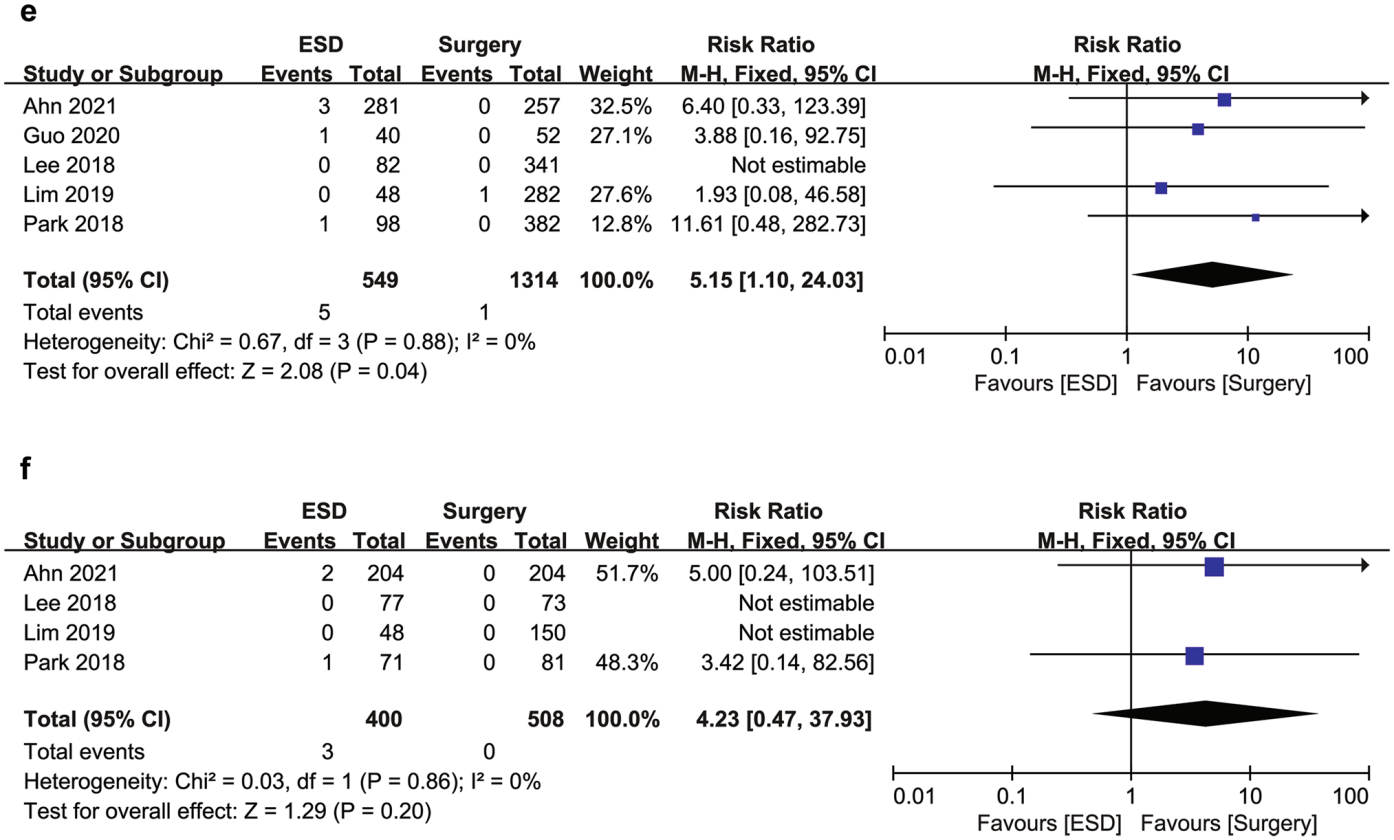
Forest plot of (**a**) recurrence in the overall cohort, (**b**) recurrence in the propensity score matching cohort, (**c**) gastric recurrence in the overall cohort, (**d**) gastric recurrence in the propensity score matching cohort, (**e**) extragastric recurrence in the overall cohort, and (**f**) extragastric recurrence in the propensity score matching cohort for endoscopic submucosal dissection and surgery for undifferentiated-type early gastric cancer. *ESD* endoscopic submucosal dissection

The ESD group showed a higher risk of recurrence than the surgery group both in the overall cohort (6.0% [33/549] vs. 0.6% [8/1314]; RR 9.17; 95% CI 4.02–20.92; *I*^2^ = 2%) and in the PSM cohort (6.3% [12/400] vs. 0.2% [1/508]; RR 12.61; 95% CI 3.43–46.37; *I*^2^ = 0%) (Fig. 4a, b). Most recurrences were gastric recurrences. Thus, the ESD group was associated with a significantly higher risk of gastric recurrence compared to the surgery group in the overall cohort (5.1% [28/549] vs. 0.5% [7/1314]; RR 8.39; 95% CI 3.66–19.22; *I*^2^ = 21%) and in the PSM cohort (5.5% [22/400] vs. 0.2% [1/508]; RR 11.25; 95% CI 3.06–41.40; *I*^2^ = 0%) (Fig. 4c, d). The ESD group also showed a significantly higher risk of extragastric recurrence compared to the surgery group in the overall cohort (0.9% [5/549] vs. 0.1% [1/1314]; RR 5.15; 95% CI 1.10–24.03; *I*^2^ = 0%) (Fig. 4e). Among the extragastric recurrences, three of five patients (0.5%) in the ESD group and one patient (0.1%) in the surgery group had a metastatic recurrence, all of whom died of gastric cancer. In particular, metastatic recurrence developed 75, 76, and 84 months after ESD, whereas it developed 31 months after surgery. The other two patients in the ESD group had LN recurrence without distant metastasis. One patient did not die of gastric cancer during the study period. However, the other patient who developed LN and local recurrence at the same time underwent salvage radical surgery but developed distant recurrence and died of gastric cancer. The increased risk of extragastric recurrence was not statistically significant in the PSM cohort (RR 4.23; 95% CI 0.47–37.93; *I*^2^ = 0%) (Fig. 4f). No heterogeneity was observed in the DFS and recurrence analyses.

### Disease-specific survival

**Fig. 5:**
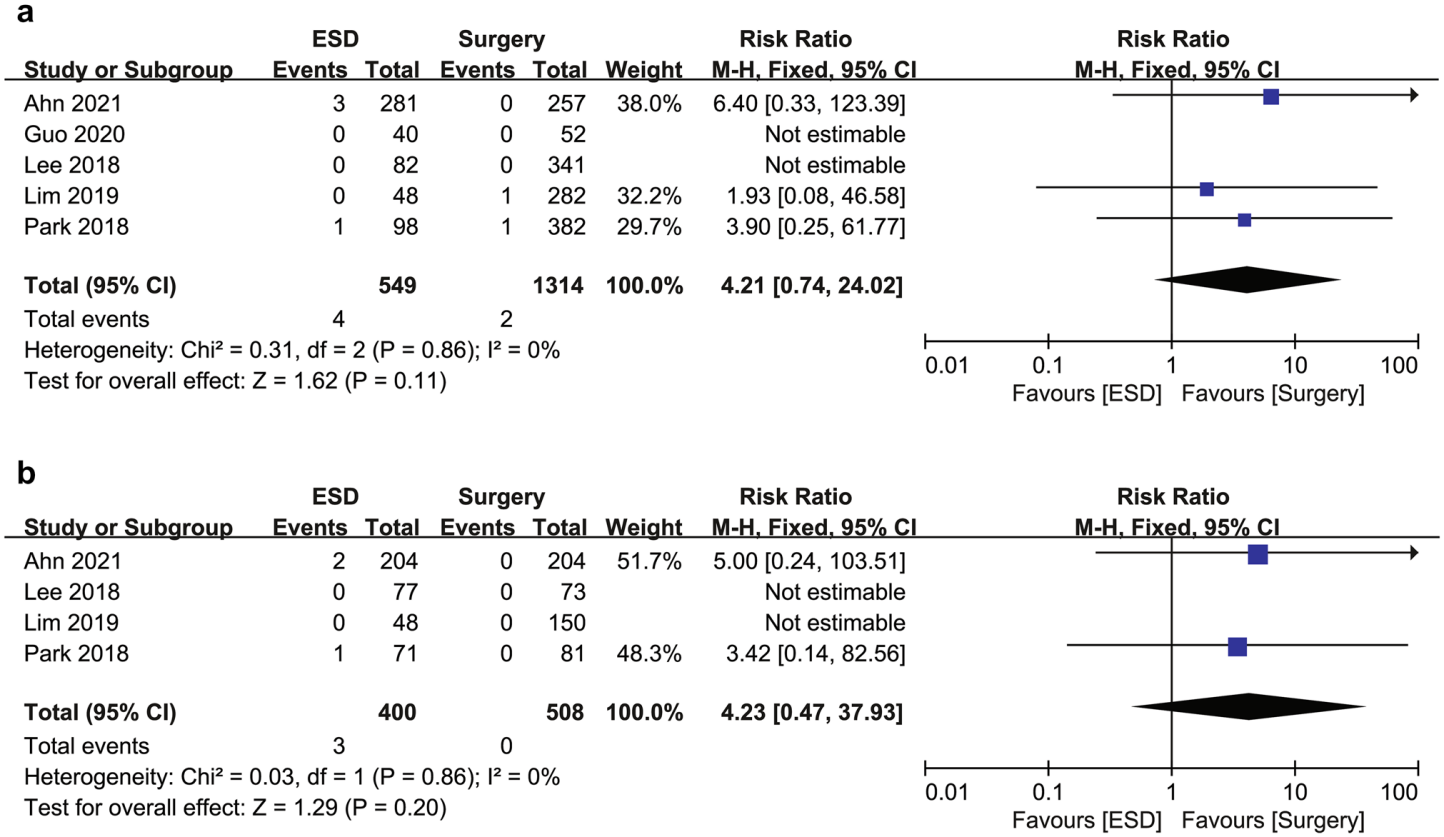
Forest plot of disease-specific survival for endoscopic submucosal dissection and surgery for undifferentiated-type early gastric cancer (**a**) in the overall cohort and (**b**) in the propensity score matching cohort. *ESD* endoscopic submucosal dissection

Overall, six patients died of gastric cancer: four in the ESD group and two in the surgery group. Three patients in the ESD group and one patient in the surgery group developed distant recurrence, while the other two developed local recurrence. DSS rates were 99.3% (545/549) in the ESD group and 99.8% (1312/1314) in the surgery group in the overall cohort (Fig. 5). Two studies were not included in the meta-analysis of DSS because no gastric cancer deaths occurred in either group. The RR for gastric cancer death was not significantly different between the ESD and surgery groups both in the overall cohort (RR 4.21; 95% CI 0.74–24.02; *I*^2^ = 0%) and in the PSM cohort (RR 4.23; 95% CI 0.47–37.93; *I*^2^ = 0%).

### Subgroup analysis and sensitivity analysis

**Fig. 6:**
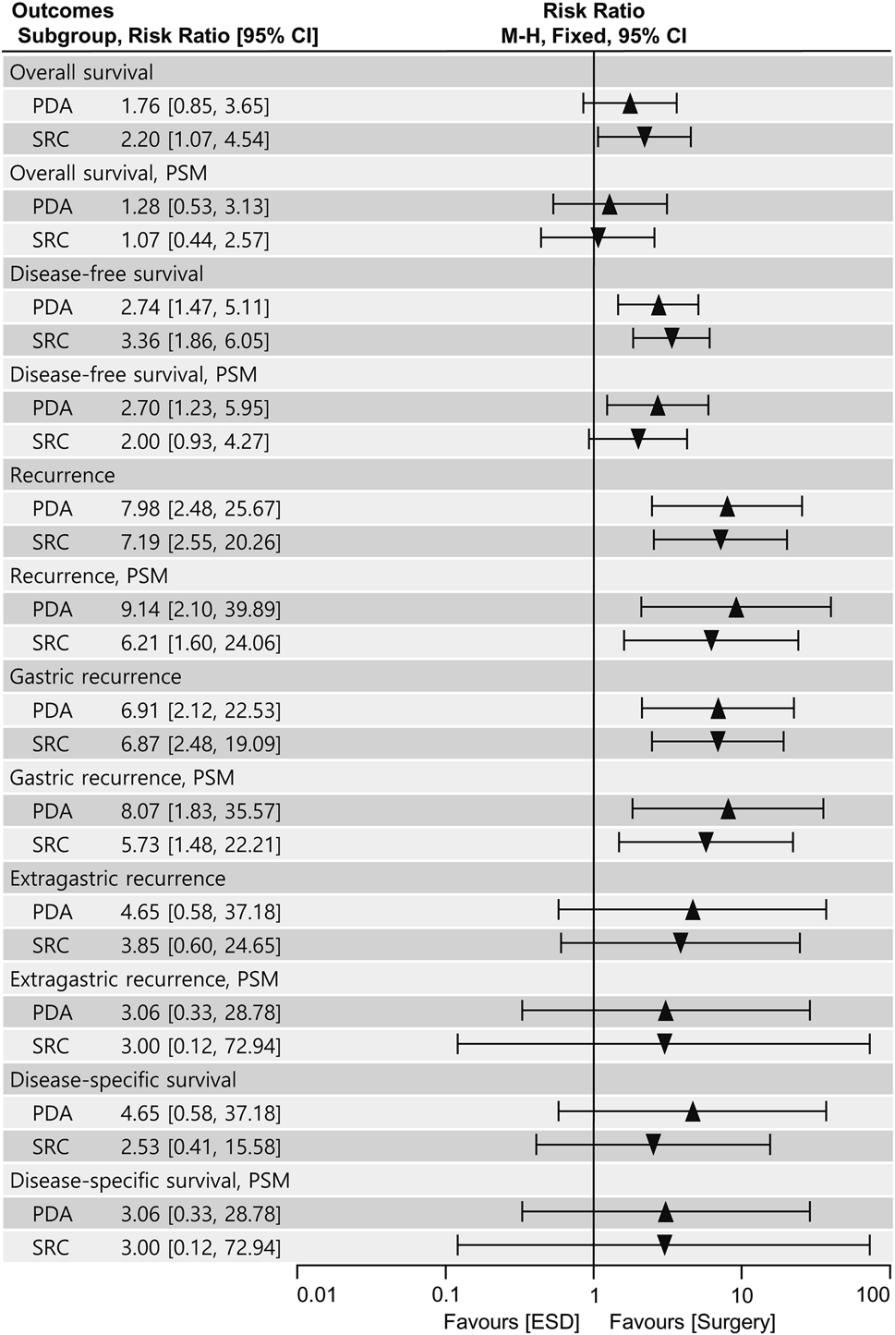
Subgroup analysis comparing poorly differentiated adenocarcinoma and signet ring cell carcinoma in the long-term outcomes between endoscopic submucosal dissection and surgery. *PDA* poorly differentiated adenocarcinoma, *SRC* signet ring cell carcinoma, *PSM* propensity score matching, *ESD* endoscopic submucosal dissection

In the subgroup analysis, there were no significant differences in the long-term outcomes associated with ESD compared with surgery between the PDA and SRC subgroups (all *P* for subgroup difference > 0.05) (Fig. 6, Supplemental Figs. 2–5). Particularly, OS was similar between the ESD and surgery groups not only in the SRC subgroup (RR 1.07; 95% CI 0.44–2.57; *I*^2^ = 0%) but also in the PDA subgroup (RR 1.28; 95% CI 0.53–3.13; *I*^2^ = 0%) of the PSM cohort (*P* for subgroup difference = 0.77). In addition, DFS was lower in the ESD group than in the surgery group for both PDA (RR 2.70; 95% CI 1.23–5.95; *I*^2^ = 0%) and SRC (RR 2.00; 95% CI 0.93–4.27; *I*^2^ = 0%) of the PSM cohort (*P* for subgroup difference = 0.59).

In the sensitivity analysis, HR was directly derived from two studies [14, 29] while being indirectly estimated in the others [13, 28, 30]. The results were consistent with those in the main analysis (Supplemental Table 3 and Figs. 6–9). The HR (95% CI *I*^2^) for OS was 2.07 (1.04–4.11, 0%) in the overall cohort and 1.15 (0.54–2.44, 0%) in the PSM cohort favoring surgery. In the PSM cohort, HR (95% CI *I*^2^) for DFS and recurrence was 2.61 (1.28–5.30, 0%) and 4.96 (0.82–29.90, 05%) favoring surgery, respectively.

### Publication bias and certainty of evidence

We generated funnel plots for the outcomes (Supplemental Figs. 10–13). Although the number of studies was limited, no publication bias was suggested.

The certainty of the evidence was very low in the overall cohort because of the very serious risk of bias and serious imprecision. In the PSM cohort, the certainty of the evidence was low because greater protection against the risk of bias was provided using PSM. However, the certainty of the evidence was still limited because of remained risk of bias and imprecision (Supplemental Tables 4–5**)**.

## Discussion

This systematic review and meta-analysis assembled data from 1863 patients who underwent curative ESD or surgery for UD EGC meeting the expanded criteria from five studies. We demonstrated that the ESD group had a worse OS than the surgery group in the overall cohort but not in the PSM cohort. However, the ESD group was associated with inferior DFS, recurrence, and gastric recurrence compared to the surgery group. The higher risk of extragastric recurrence associated with ESD was significant in the overall cohort but not in the PSM cohort. Moreover, DSS did not differ between the two groups. In addition, there were no significant subgroup differences between PDA and SRC in the long-term outcomes associated with ESD in comparison with surgery.

In our study, we found that ESD was associated with inferior OS to surgery in the overall cohort but not in the PSM cohort. These results are consistent with the results of the most recent and largest cohort study [14]. Prior studies suggested no significant difference in the OS between ESD and surgery both before and after PSM, which indicates the possible low study power of these studies [13, 28–30]. The difference in OS between the overall and PSM cohorts in our study may be explained by the differences in age and underlying illness because these differences were considerably attenuated after PSM. This is also supported by the fact that gastric cancer deaths were rare in both groups and that DSS was not significantly different in both the overall and PSM cohorts. The OS in our study was lower than the results from a recent Japanese prospective study (5 years OS, 99.3%) [4]. This may be because the prospective study included patients with good performance status (ECOG 0 and 1) and excluded those with severe underlying illness. Our results are also consistent with recent systematic reviews that compared ESD or endoscopic resection with surgery for EGC meeting the expanded criteria [17–20]. Therefore, our study suggests that the OS after curative ESD may be comparable to that after surgery for UD EGC.

In our meta-analysis, extragastric recurrence occurred more frequently after ESD than after surgery, although this did not compromise DSS in the ESD group. However, this result should be interpreted along with a few other points. First, although it was not evaluated in our study, radical gastrectomy was associated with an approximately twofold higher risk of postoperative complications compared to endoscopic resection or ESD [17, 19]. Second, in a multicenter prospective study with central pathology review, there was no extragastric recurrence for 5 years after curative ESD for UD EGC [4]. Because our study was a meta-analysis of retrospective cohort studies, it was not possible to conduct a pathological review for potential pathological misclassification. Nevertheless, three patients in the ESD group and one patient in the surgery group with distant recurrence resulted in gastric cancer deaths. In particular, distant recurrences in the ESD group developed after 5 years of follow-up (75, 76, and 84 months). A previous study suggested that recurrence of primary cancer seemed to develop more slowly after ESD for EGC than after surgery for advanced gastric cancer [31]. Another study that investigated long-term outcomes after noncurative ESD for EGC also suggested a need for long-term follow-up data [32]. For example, the Dutch trial that compared D1 versus D2 dissection for gastric cancer followed patients for a median of 15.2 years [33]. Therefore, further prospective long-term follow-up studies are necessary to verify the risk of late extragastric recurrence after curative ESD for UD EGC.

The ESD group was associated with inferior DFS and higher recurrence and gastric recurrence compared to the surgery group in our study. The lower DFS and higher recurrence in the ESD group were because of a higher rate of gastric recurrence rather than extragastric recurrence. The gastric recurrence comprised the majority of the overall recurrence and occurred 8.4 times more frequently in the ESD group than in the surgery group. This was because gastric mucosa was preserved in ESD and from which metachronous gastric cancer developed. A previous study reported the risk of metachronous recurrence was 6.7 times higher after endoscopic resection compared to surgery for EGC [34]. Our results are consistent with this report and previous systematic reviews [17–20]. It was reported that UD EGC was associated with a lower risk of metachronous recurrence compared to differentiated-type EGC [35, 36]. A recent post hoc analysis of a prospective study reported that the 5-year cumulative incidence of metachronous recurrence was 1.0% [37]. In contrast, UD EGC has been suggested as a risk factor for local recurrence after complete endoscopic resection [38]. Scheduled regular surveillance endoscopy after ESD was suggested to help detection of recurrence at an early stage enough for curative resection [39]. Therefore, endoscopy surveillance may still be important for UD EGC after curative ESD.

Interestingly, in our study, the long-term outcomes associated with ESD compared with surgery were not affected by the histologic subtypes of PDA and SRC. It has been hypothesized that PDA might be associated with a higher risk of distant metastasis or mortality compared with SRC after curative ESD [13, 14]. However, we observed similar numbers of extragastric recurrences and gastric cancer deaths between the PDA and SRC subgroups. Thus, there was insufficient evidence in our study to suggest that ESD indications for UD EGC should be different between PDA and SRC.

Our study has several limitations. First, the included studies were retrospective cohort studies. Although four of the five studies performed PSM analysis, the lack of prospective cohort studies or RCTs might have led to an inevitable selection bias. Second, we used RR instead of HR as an effect measure because HR was available only in two of the five studies. However, in the sensitivity analysis where some HRs were indirectly estimated, the results were consistent with the main analysis using OR. Third, the included studies were conducted in Korea and China. Because there were no Western studies, the current results may not be directly applicable to patients or clinical settings in Western countries. Fourth, we excluded several studies that included both differentiated-type EGC and UD EGC but did not provide subgroup analysis data for UD EGC. Therefore, the data from these studies may have been underrepresented, leading to a potential publication bias. Lastly, there may be clinical heterogeneity, especially in pathological tissue handling between ESD and surgical specimens. In particular, section intervals are wider for surgical specimens (5–7 mm) than for ESD specimens (2–3 mm) [1, 2, 25], which might have led to the underestimation of submucosal invasion or lymphovascular invasion in the surgical specimens [40]. Thus, the risk factor assessment for extragastric recurrence might not be comparable between the ESD and surgery groups even after PSM, and our long-term outcome data should be interpreted with caution and validated in further prospective studies.

In conclusion, OS after curative ESD for UD EGC meeting the expanded criteria was not significantly different from that after surgery in the PSM cohort. DSS was not significantly different between the ESD and surgery groups. However, DFS and recurrence were inferior after ESD than after surgery. In addition, extragastric recurrences that led to gastric cancer deaths occurred in both the ESD and surgery groups. Therefore, more evidence is needed, a longer period of observation, further matched cohort studies, and eventually, prospective RCTs with longer and standardized follow-up.

## Supplementary Information

Below is the link to the electronic supplementary material.

Supplementary file1 (DOCX 2896 kb)

## Declarations

### Data availability

The meta-analysis data are not publically available because the investigators of the review are not owners of individual participant data.

## References

[1] Ono H, Yao K, Fujishiro M, Oda I, Uedo N, Nimura S, Yahagi N, Iishi H, Oka M, Ajioka Y, Fujimoto K (2021). Guidelines for endoscopic submucosal dissection and endoscopic mucosal resection for early gastric cancer (second edition). Dig Endosc.

[2] Japanese Gastric Cancer Association (2020) Japanese gastric cancer treatment guidelines 2018 (5th edition). Gastric Cancer. 10.1007/s10120-020-01042-y 10.1007/s10120-020-01042-y PMC7790804 32060757

[3] Guideline Committee of the Korean Gastric Cancer Association, Development Working Group, Review Panel (2019). Korean practice guideline for gastric cancer 2018: an evidence-based, multi-disciplinary approach. J Gastric Cancer.

[4] Takizawa K, Ono H, Hasuike N, Takashima A, Minashi K, Boku N, Kushima R, Katayama H, Ogawa G, Fukuda H, Fujisaki J, Oda I, Yano T, Hori S, Doyama H, Hirasawa K, Yamamoto Y, Ishihara R, Tanabe S, Niwa Y, Nakagawa M, Terashima M, Muto M, Gastrointestinal Endoscopy G, the Stomach Cancer Study Group of Japan Clinical Oncology G (2021). A nonrandomized, single-arm confirmatory trial of expanded endoscopic submucosal dissection indication for undifferentiated early gastric cancer: Japan Clinical Oncology Group study (JCOG1009/1010). Gastric Cancer.

[5] Hirasawa T, Gotoda T, Miyata S, Kato Y, Shimoda T, Taniguchi H, Fujisaki J, Sano T, Yamaguchi T (2009). Incidence of lymph node metastasis and the feasibility of endoscopic resection for undifferentiated-type early gastric cancer. Gastric Cancer.

[6] Lee IS, Lee S, Park YS, Gong CS, Yook JH, Kim BS (2017). Applicability of endoscopic submucosal dissection for undifferentiated early gastric cancer: Mixed histology of poorly differentiated adenocarcinoma and signet ring cell carcinoma is a worse predictive factor of nodal metastasis. Surg Oncol.

[7] Yoon HJ, Kim YH, Kim JH, Kim H, Kim H, Park JJ, Youn YH, Park H, Kim JW, Hyung WJ, Noh SH, Choi SH (2016). Are new criteria for mixed histology necessary for endoscopic resection in early gastric cancer?. Pathol Res Pract.

[8] Lee JH, Choi MG, Min BH, Noh JH, Sohn TS, Bae JM, Kim S (2012). Predictive factors for lymph node metastasis in patients with poorly differentiated early gastric cancer. Br J Surg.

[9] Pessorrusso FCS, Felipe-Silva A, Jacob CE, Ramos M, Ferreira VAA, de Mello ES, Zilberstein B, Ribeiro U, Maluf-Filho F (2018). Risk assessment of lymph node metastases in early gastric adenocarcinoma fulfilling expanded endoscopic resection criteria. Gastrointest Endosc.

[10] Odagaki T, Suzuki H, Oda I, Yoshinaga S, Nonaka S, Katai H, Taniguchi H, Kushima R, Saito Y (2013). Small undifferentiated intramucosal gastric cancer with lymph-node metastasis: case report. World J Gastroenterol.

[11] Nasu J, Hori S, Asagi A, Nishina T, Ikeda Y, Tanimizu M, Iguchi H, Aogi K, Kurita A, Nishimura R (2010). A case of small undifferentiated intramucosal gastric cancer with lymph node metastasis. Gastric Cancer.

[12] Hirasawa T, Fujisaki J, Fukunaga T, Yamamoto Y, Yamaguchi T, Katori M, Yamamoto N (2010). Lymph node metastasis from undifferentiated-type mucosal gastric cancer satisfying the expanded criteria for endoscopic resection based on routine histological examination. Gastric Cancer.

[13] Park JC, Lee YK, Kim SY, Roh Y, Hahn KY, Shin SK, Lee SK, Lee YC, Kim HI, Cheong JH, Hyung WJ, Noh SH (2018). Long-term outcomes of endoscopic submucosal dissection in comparison to surgery in undifferentiated-type intramucosal gastric cancer using propensity score analysis. Surg Endosc.

[14] Ahn JY, Kim YI, Shin WG, Yang HJ, Nam SY, Min BH, Jang JY, Lim JH, Kim J, Lee WS, Lee BE, Joo MK, Park JM, Lee HL, Gweon TG, Park MI, Choi J, Tae CH, Kim YW, Park B, Choi IIJ (2021). Comparison between endoscopic submucosal resection and surgery for the curative resection of undifferentiated-type early gastric cancer within expanded indications: a nationwide multi-center study. Gastric Cancer.

[15] Zhao X, Cai A, Xi H, Chen L, Peng Z, Li P, Liu N, Cui J, Li H (2017). Predictive factors for lymph node metastasis in undifferentiated early gastric cancer: a systematic review and meta-analysis. J Gastrointest Surg.

[16] Liu Q, Ding L, Qiu X, Meng F (2020). Updated evaluation of endoscopic submucosal dissection versus surgery for early gastric cancer: a systematic review and meta-analysis. Int J Surg.

[17] Li H, Feng LQ, Bian YY, Yang LL, Liu DX, Huo ZB, Zeng L (2019). Comparison of endoscopic submucosal dissection with surgical gastrectomy for early gastric cancer: an updated meta-analysis. World J Gastrointest Oncol.

[18] Gu L, Khadaroo PA, Chen L, Li X, Zhu H, Zhong X, Pan J, Chen M (2019). Comparison of long-term outcomes of endoscopic submucosal dissection and surgery for early gastric cancer: a systematic review and meta-analysis. J Gastrointest Surg.

[19] An L, Gaowa S, Cheng H, Hou M (2019). Long-term outcomes comparison of endoscopic resection with gastrectomy for treatment of early gastric cancer: a systematic review and meta-analysis. Front Oncol.

[20] Abdelfatah MM, Barakat M, Ahmad D, Ibrahim M, Ahmed Y, Kurdi Y, Grimm IS, Othman MO (2019). Long-term outcomes of endoscopic submucosal dissection versus surgery in early gastric cancer: a systematic review and meta-analysis. Eur J Gastroenterol Hepatol.

[21] Huh CW, Ma DW, Kim BW, Kim JS, Lee SJ (2021). Endoscopic submucosal dissection versus surgery for undifferentiated-type early gastric cancer: a systematic review and meta-analysis. Clin Endosc.

[22] Page MJ, McKenzie JE, Bossuyt PM, Boutron I, Hoffmann TC, Mulrow CD, Shamseer L, Tetzlaff JM, Akl EA, Brennan SE, Chou R, Glanville J, Grimshaw JM, Hrobjartsson A, Lalu MM, Li T, Loder EW, Mayo-Wilson E, McDonald S, McGuinness LA, Stewart LA, Thomas J, Tricco AC, Welch VA, Whiting P, Moher D (2021). The PRISMA 2020 statement: an updated guideline for reporting systematic reviews. BMJ.

[23] Sterne JA, Hernan MA, Reeves BC, Savovic J, Berkman ND, Viswanathan M, Henry D, Altman DG, Ansari MT, Boutron I, Carpenter JR, Chan AW, Churchill R, Deeks JJ, Hrobjartsson A, Kirkham J, Juni P, Loke YK, Pigott TD, Ramsay CR, Regidor D, Rothstein HR, Sandhu L, Santaguida PL, Schunemann HJ, Shea B, Shrier I, Tugwell P, Turner L, Valentine JC, Waddington H, Waters E, Wells GA, Whiting PF, Higgins JP (2016). ROBINS-I: a tool for assessing risk of bias in non-randomised studies of interventions. BMJ.

[24] McGuinness LA, Higgins JPT (2021). Risk-of-bias VISualization (robvis): an R package and Shiny web app for visualizing risk-of-bias assessments. Res Synth Methods.

[25] Japanese Gastric Cancer Association (2011) Japanese classification of gastric carcinoma: 3rd English edition. Gastric Cancer 14:101–112. 10.1007/s10120-011-0041-5 10.1007/s10120-011-0041-5 21573743

[26] Tierney JF, Stewart LA, Ghersi D, Burdett S, Sydes MR (2007). Practical methods for incorporating summary time-to-event data into meta-analysis. Trials.

[27] Balshem H, Helfand M, Schunemann HJ, Oxman AD, Kunz R, Brozek J, Vist GE, Falck-Ytter Y, Meerpohl J, Norris S, Guyatt GH (2011). GRADE guidelines: 3. Rating the quality of evidence. J Clin Epidemiol.

[28] Guo A, Du C, Tian S, Sun L, Guo M, Lu L, Peng L (2020). Long-term outcomes of endoscopic submucosal dissection versus surgery for treating early gastric cancer of undifferentiated-type. Medicine (Baltimore).

[29] Lim JH, Kim J, Kim SG, Chung H (2019). Long-term clinical outcomes of endoscopic vs. surgical resection for early gastric cancer with undifferentiated histology. Surg Endosc.

[30] Lee S, Choi KD, Han M, Na HK, Ahn JY, Jung KW, Lee JH, Kim DH, Song HJ, Lee GH, Yook JH, Kim BS, Jung HY (2018). Long-term outcomes of endoscopic submucosal dissection versus surgery in early gastric cancer meeting expanded indication including undifferentiated-type tumors: a criteria-based analysis. Gastric Cancer.

[31] Min BH, Kim ER, Kim KM, Park CK, Lee JH, Rhee PL, Kim JJ (2015). Surveillance strategy based on the incidence and patterns of recurrence after curative endoscopic submucosal dissection for early gastric cancer. Endoscopy.

[32] Hatta W, Gotoda T, Oyama T, Kawata N, Takahashi A, Yoshifuku Y, Hoteya S, Nakamura K, Hirano M, Esaki M, Matsuda M, Ohnita K, Shimoda R, Yoshida M, Dohi O, Takada J, Tanaka K, Yamada S, Tsuji T, Ito H, Hayashi Y, Nakamura T, Shimosegawa T (2017). Is radical surgery necessary in all patients who do not meet the curative criteria for endoscopic submucosal dissection in early gastric cancer? A multi-center retrospective study in Japan. J Gastroenterol.

[33] Songun I, Putter H, Kranenbarg EM, Sasako M, van de Velde CJ (2010). Surgical treatment of gastric cancer: 15-year follow-up results of the randomised nationwide Dutch D1D2 trial. Lancet Oncol.

[34] Choi KS, Jung HY, Choi KD, Lee GH, Song HJ, Kim do H, Lee JH, Kim MY, Kim BS, Oh ST, Yook JH, Jang SJ, Yun SC, Kim SO, Kim JH (2011). EMR versus gastrectomy for intramucosal gastric cancer: comparison of long-term outcomes. Gastrointest Endosc.

[35] Ishioka M, Yoshio T, Miyamoto Y, Namikawa K, Tokai Y, Yoshimizu S, Horiuchi Y, Ishiyama A, Hirasawa T, Tsuchida T, Fujisaki J (2021). Incidence of metachronous cancer after endoscopic submucosal dissection: a comparison between undifferentiated-type and differentiated-type early gastric cancer. Gastrointest Endosc.

[36] Park CH, Kim EH, Kang JH, Chung H, Park JC, Shin SK, Lee SK, Lee YC (2016). Low incidence of synchronous or metachronous tumors after endoscopic submucosal dissection for early gastric cancer with undifferentiated histology. PLoS ONE.

[37] Abe S, Takizawa K, Oda I, Mizusawa J, Kadota T, Ono H, Hasuike N, Yano T, Yamamoto Y, Horiuchi Y, Nagata S, Yoshikawa T, Terashima M, Muto M (2021). Incidence and treatment outcomes of metachronous gastric cancer occurring after curative endoscopic submucosal dissection of undifferentiated-type early gastric cancer: Japan Clinical Oncology Group study-post hoc analysis of JCOG1009/1010. Gastric Cancer.

[38] Yun GW, Kim JH, Lee YC, Lee SK, Shin SK, Park JC, Chung HS, Park JJ, Youn YH, Park H (2015). What are the risk factors for residual tumor cells after endoscopic complete resection in gastric epithelial neoplasia?. Surg Endosc.

[39] Hahn KY, Park JC, Kim EH, Shin S, Park CH, Chung H, Shin SK, Lee SK, Lee YC (2016). Incidence and impact of scheduled endoscopic surveillance on recurrence after curative endoscopic resection for early gastric cancer. Gastrointest Endosc.

[40] Kim YI, Kook MC, Choi JE, Lee JY, Kim CG, Eom BW, Yoon HM, Ryu KW, Kim YW, Choi IJ (2020). Evaluation of submucosal or lymphovascular invasion detection rates in early gastric cancer based on pathology section interval. J Gastric Cancer.

