# Supplementary material for: Comparison of long-term outcomes of endoscopic submucosal dissection and surgery for undifferentiated-type early gastric cancer meeting the expanded criteria: a systematic review and meta-analysis
Source: Surg Endosc. 2022 Feb 22;36(6):3686–97. doi: 10.1007/s00464-022-09126-9 (PMC9085673; doi:10.1007/s00464-022-09126-9)
Supplement: Supplementary file 1 — Supplementary file1 (DOCX 2896 kb) [file 464_2022_9126_MOESM1_ESM.docx]

Supplementary Information Table of Contents

[**Supplementary Table 1** Detailed search strategies for the PubMed, Embase, and Cochrane Library databases. 3](#_Toc92554015)

[**Supplementary Table 2** Characteristics of studies excluded in the full-text review. 9](#_Toc92554016)

[**Supplementary Table 3** Sensitivity analysis using hazard ratio as a measure of outcome 12](#_Toc92554017)

[**Supplementary Table 4** GRADE evidence table in the overall cohort 13](#_Toc92554018)

[**Supplementary Table 5** GRADE evidence table in the propensity score matching cohort 15](#_Toc92554019)

[**Supplementary Fig. 1** Risk of bias of studies included in the meta-analysis. 17](#_Toc92554020)

[**Supplementary Fig. 2** Subgroup analysis comparing poorly differentiated adenocarcinoma and signet ring cell carcinoma in overall survival between endoscopic submucosal dissection and surgery (**a**) in the overall cohort and (**b**) in the propensity score matching cohort. 19](#_Toc92554021)

[**Supplementary Fig. 3** Subgroup analysis comparing poorly differentiated adenocarcinoma and signet ring cell carcinoma in disease-free survival between endoscopic submucosal dissection and surgery (**a**) in the overall cohort and (**b**) in the propensity score matching cohort. 20](#_Toc92554022)

[**Supplementary Fig. 4** Subgroup analysis comparing poorly differentiated adenocarcinoma and signet ring cell carcinoma in (**a**) recurrence in the overall cohort, (**b**), recurrence in the propensity score matching cohort, (**c**) gastric recurrence in the overall cohort, (**d**) gastric recurrence in the propensity score matching cohort, (**e**) extragastric recurrence in the overall cohort, and (**f**) extragastric recurrence in the propensity score matching cohort between endoscopic submucosal dissection and surgery. 21](#_Toc92554023)

[**Supplementary Fig. 5** Subgroup analysis comparing poorly differentiated adenocarcinoma and signet ring cell carcinoma in disease-specific survival between endoscopic submucosal dissection and surgery (**a**) in the overall cohort and (**b**) in the propensity score matching cohort. 24](#_Toc92554024)

[**Supplementary Fig. 6** Sensitivity analysis using hazard ratio as a measure of outcome in overall survival between endoscopic submucosal dissection and surgery (**a**) in the overall cohort and (**b**) in the propensity score matching cohort. 25](#_Toc92554025)

[**Supplementary Fig. 7** Sensitivity analysis using hazard ratio as a measure of outcome in disease-free survival between endoscopic submucosal dissection and surgery (**a**) in the overall cohort and (**b**) in the propensity score matching cohort. 26](#_Toc92554026)

[**Supplementary Fig. 8** Sensitivity analysis using hazard ratio as a measure of outcome in (**a**) recurrence in the overall cohort, (**b**), recurrence in the propensity score matching cohort, (**c**) gastric recurrence in the overall cohort, (**d**) gastric recurrence in the propensity score matching cohort, (**e**) extragastric recurrence in the overall cohort, and (**f**) extragastric recurrence in the propensity score matching cohort between endoscopic submucosal dissection and surgery. 27](#_Toc92554027)

[**Supplementary Fig. 9** Sensitivity analysis using hazard ratio as a measure of outcome in disease-specific survival between endoscopic submucosal dissection and surgery (**a**) in the overall cohort and (**b**) in the propensity score matching cohort. 29](#_Toc92554028)

[**Supplementary Fig. 10** Funnel plots for overall survival (**a**) in the overall cohort and (**b**) in the propensity score matching cohort. 30](#_Toc92554029)

[**Supplementary Fig. 11** Funnel plots for disease-free survival (**a**) in the overall cohort and (**b**) in the propensity score matching cohort. 31](#_Toc92554030)

[**Supplementary Fig. 12** Funnel plots for (**a**) recurrence in the overall cohort, (**b**), recurrence in the propensity score matching cohort, (**c**) gastric recurrence in the overall cohort, (**d**) gastric recurrence in the propensity score matching cohort, (**e**) extragastric recurrence in the overall cohort, and (**f**) extragastric recurrence in the propensity score matching cohort. 32](#_Toc92554031)

[**Supplementary Fig. 13** Funnel plots for disease-specific survival (**a**) in the overall cohort and (**b**) in the propensity score matching cohort. 35](#_Toc92554032)

[**References** 36](#_Toc92554033)

# **Supplementary Table 1** Detailed search strategies for the PubMed, Embase, and Cochrane Library databases.

| A: Search strategy for PubMed | | |
| --- | --- | --- |
| # | Query | Results |
| 1 | ‘‘early gastric cancer’’[Title/Abstract] OR "EGC"[Title/Abstract] OR ‘‘early gastric neoplasm’’[Title/Abstract] OR ‘‘early stomach cancer’’[Title/Abstract] OR ‘‘early stomach neoplasm’’[Title/Abstract] | 6,213 |
| 2 | "Stomach Neoplasms"[Mesh] | 98,719 |
| 3 | ‘‘early gastric cancer’’[Title/Abstract] OR "EGC"[Title/Abstract] OR ‘‘early gastric neoplasm’’[Title/Abstract] OR ‘‘early stomach cancer’’[Title/Abstract] OR ‘‘early stomach neoplasm’’[Title/Abstract] OR "Stomach Neoplasms"[Mesh] | 100,756 |
| 4 | ‘‘endoscopic submucosal dissection’’[Title/Abstract] OR "ESD"[Title/Abstract] OR ‘‘endoscopic resection’’[Title/Abstract] OR "ER"[Title/Abstract] | 106,791 |
| 5 | "Endoscopic Mucosal Resection"[Mesh] | 1,958 |
| 6 | (‘‘endoscopic submucosal dissection’’[Title/Abstract] OR "ESD"[Title/Abstract] OR ‘‘endoscopic resection’’[Title/Abstract] OR "ER"[Title/Abstract]) OR ("Endoscopic Mucosal Resection"[Mesh]) | 107,318 |
| 7 | ‘‘gastrectomy’’[Title/Abstract] OR ‘‘surgery’’[Title/Abstract] OR "resection surgery"[Title/Abstract] OR "operation"[Title/Abstract] | 1,560,262 |
| 8 | "Gastrectomy"[Mesh] | 36,336 |
| 9 | (‘‘gastrectomy’’[Title/Abstract] OR ‘‘surgery’’[Title/Abstract] OR "resection surgery"[Title/Abstract] OR "operation"[Title/Abstract]) OR ("Gastrectomy"[Mesh]) | 1,570,509 |
| 10 | ((‘‘early gastric cancer’’[Title/Abstract] OR "EGC"[Title/Abstract] OR ‘‘early gastric neoplasm’’[Title/Abstract] OR ‘‘early stomach cancer’’[Title/Abstract] OR ‘‘early stomach neoplasm’’[Title/Abstract] OR "Stomach Neoplasms"[Mesh]) AND ((‘‘endoscopic submucosal dissection’’[Title/Abstract] OR "ESD"[Title/Abstract] OR ‘‘endoscopic resection’’[Title/Abstract] OR "ER"[Title/Abstract]) OR ("Endoscopic Mucosal Resection"[Mesh]))) AND ((‘‘gastrectomy’’[Title/Abstract] OR ‘‘surgery’’[Title/Abstract] OR "resection surgery"[Title/Abstract] OR "operation"[Title/Abstract]) OR ("Gastrectomy"[Mesh])) | 1,129 |
| 11 | "Randomized Controlled Trial" [Publication Type] OR "Clinical Trial" [Publication Type] OR "Clinical Trials as Topic"[Mesh] OR Randomized[Title/Abstract] OR Randomised[Title/Abstract] OR Randomization[Title/Abstract] OR Randomisation[Title/Abstract] OR "Random Allocation"[Title/Abstract] OR "Double Blind Method"[Title/Abstract] OR "Double-Blind Method"[Mesh]OR "Single Blind Method"[Title/Abstract] OR "Single-Blind Method"[Mesh] OR "clinical trial"[Title/Abstract] OR "multicenter study"[Title/Abstract] OR "Multicenter Study" [Publication Type] OR "Multicenter Studies as Topic"[Mesh] OR placebo[Title/Abstract] OR "Placebos"[Mesh] OR "randomly allocated"[Title/Abstract] OR "Observational Study" [Title/Abstract] OR Observational[Title/Abstract] OR "Observational Study" [Publication Type] OR "Observational Studies as Topic"[Mesh] OR "Retrospective Studies"[Mesh] OR "Retrospective studies"[Title/Abstract] OR "Epidemiologic Studies"[Title/Abstract] OR "Epidemiologic Studies"[Mesh] sss | 4,014,634 |
| 12 | (((‘‘early gastric cancer’’[Title/Abstract] OR "EGC"[Title/Abstract] OR ‘‘early gastric neoplasm’’[Title/Abstract] OR ‘‘early stomach cancer’’[Title/Abstract] OR ‘‘early stomach neoplasm’’[Title/Abstract] OR "Stomach Neoplasms"[Mesh]) AND ((‘‘endoscopic submucosal dissection’’[Title/Abstract] OR "ESD"[Title/Abstract] OR ‘‘endoscopic resection’’[Title/Abstract] OR "ER"[Title/Abstract]) OR ("Endoscopic Mucosal Resection"[Mesh]))) AND ((‘‘gastrectomy’’[Title/Abstract] OR ‘‘surgery’’[Title/Abstract] OR "resection surgery"[Title/Abstract] OR "operation"[Title/Abstract]) OR ("Gastrectomy"[Mesh]))) AND ("Randomized Controlled Trial" [Publication Type] OR "Clinical Trial" [Publication Type] OR "Clinical Trials as Topic"[Mesh] OR Randomized[Title/Abstract] OR Randomised[Title/Abstract] OR Randomization[Title/Abstract] OR Randomisation[Title/Abstract] OR "Random Allocation"[Title/Abstract] OR "Double Blind Method"[Title/Abstract] OR "Double-Blind Method"[Mesh]OR "Single Blind Method"[Title/Abstract] OR "Single-Blind Method"[Mesh] OR "clinical trial"[Title/Abstract] OR "multicenter study"[Title/Abstract] OR "Multicenter Study" [Publication Type] OR "Multicenter Studies as Topic"[Mesh] OR placebo[Title/Abstract] OR "Placebos"[Mesh] OR "randomly allocated"[Title/Abstract] OR "Observational Study" [Title/Abstract] OR Observational[Title/Abstract] OR "Observational Study" [Publication Type] OR "Observational Studies as Topic"[Mesh] OR "Retrospective Studies"[Mesh] OR "Retrospective studies"[Title/Abstract] OR "Epidemiologic Studies"[Title/Abstract] OR "Epidemiologic Studies"[Mesh]) | 578 |
| 13 | (((‘‘early gastric cancer’’[Title/Abstract] OR "EGC"[Title/Abstract] OR ‘‘early gastric neoplasm’’[Title/Abstract] OR ‘‘early stomach cancer’’[Title/Abstract] OR ‘‘early stomach neoplasm’’[Title/Abstract] OR "Stomach Neoplasms"[Mesh]) AND ((‘‘endoscopic submucosal dissection’’[Title/Abstract] OR "ESD"[Title/Abstract] OR ‘‘endoscopic resection’’[Title/Abstract] OR "ER"[Title/Abstract]) OR ("Endoscopic Mucosal Resection"[Mesh]))) AND ((‘‘gastrectomy’’[Title/Abstract] OR ‘‘surgery’’[Title/Abstract] OR "resection surgery"[Title/Abstract] OR "operation"[Title/Abstract]) OR ("Gastrectomy"[Mesh]))) AND ("Randomized Controlled Trial" [Publication Type] OR "Clinical Trial" [Publication Type] OR "Clinical Trials as Topic"[Mesh] OR Randomized[Title/Abstract] OR Randomised[Title/Abstract] OR Randomization[Title/Abstract] OR Randomisation[Title/Abstract] OR "Random Allocation"[Title/Abstract] OR "Double Blind Method"[Title/Abstract] OR "Double-Blind Method"[Mesh]OR "Single Blind Method"[Title/Abstract] OR "Single-Blind Method"[Mesh] OR "clinical trial"[Title/Abstract] OR "multicenter study"[Title/Abstract] OR "Multicenter Study" [Publication Type] OR "Multicenter Studies as Topic"[Mesh] OR placebo[Title/Abstract] OR "Placebos"[Mesh] OR "randomly allocated"[Title/Abstract] OR "Observational Study" [Title/Abstract] OR Observational[Title/Abstract] OR "Observational Study" [Publication Type] OR "Observational Studies as Topic"[Mesh] OR "Retrospective Studies"[Mesh] OR "Retrospective studies"[Title/Abstract] OR "Epidemiologic Studies"[Title/Abstract] OR "Epidemiologic Studies"[Mesh]) Filters: Humans | 567 |
| B: Search strategy for Embase | | |
| # | Query | Results |
| 1 | ‘early gastric cancer':ti,ab OR 'EGC':ti,ab OR 'early gastric neoplasm':ti,ab OR 'early stomach cancer':ti,ab OR 'early stomach neoplasm':ti,ab | 9,332 |
| 2 | stomach tumor'/exp | 170,456 |
| 3 | #1 OR #2 | 172,089 |
| 4 | endoscopic submucosal dissection':ti,ab OR 'ESD':ti,ab OR ‘endoscopic mucosal resection':ti,ab OR ‘EMR':ti,ab OR ‘endoscopic resection':ti,ab OR 'ER':ti,ab | 178,494 |
| 5 | endoscopic mucosal resection'/exp | 7,038 |
| 6 | #4 OR #5 | 180,262 |
| 7 | gastrectomy':ti,ab OR ‘surgery':ti,ab OR 'resection surgery':ti,ab OR 'operation':ti,ab | 2,057,855 |
| 8 | gastrectomy'/exp | 67,303 |
| 9 | #7 OR #8 | 2,077,284 |
| 10 | Randomized':ti,ab OR 'Randomised':ti,ab OR 'Randomization':ti,ab OR 'Randomisation':ti,ab OR 'Random Allocation':ti,ab OR 'Double Blind Method':ti,ab OR 'Single Blind Method':ti,ab OR 'clinical trial':ti,ab OR 'multicenter study':ti,ab OR 'placebo':ti,ab OR 'randomly allocated':ti,ab OR 'Observational Study':ti,ab OR 'Observational':ti,ab OR 'Retrospective studies':ti,ab OR 'Epidemiologic Studies':ti,ab | 1,573,634 |
| 11 | randomized controlled trial'/exp OR 'clinical trial'/exp OR 'clinical trial (topic)'/exp OR 'double blind procedure'/exp OR 'single blind procedure'/exp OR 'multicenter study'/exp OR 'multicenter study (topic)'/exp OR 'placebo'/exp OR 'observational study'/exp OR 'retrospective study'/exp | 3,212,187 |
| 12 | 10 OR 11 | 3,692,660 |
| 13 | #3 AND #6 AND #9 AND 12 AND [humans]/lim | 843 |
| C: Search strategy for Cochrane Library | | |
| # | Query | Results |
| 1 | (‘‘early gastric cancer’’ OR "EGC" OR ‘‘early gastric neoplasm’’ OR ‘‘early stomach cancer’’ OR ‘‘early stomach neoplasm’’):ti,ab,kw | 1350 |
| 2 | MeSH descriptor: [Stomach Neoplasms] explode all trees | 2621 |
| 3 | #1 OR #2 | 3632 |
| 4 | (‘‘endoscopic submucosal dissection’’ OR "ESD" OR ‘‘endoscopic mucosal resection’’ OR ‘‘EMR’’ OR ‘‘endoscopic resection’’ OR "ER"):ti,ab,kw | 11132 |
| 5 | MeSH descriptor: [Endoscopic Mucosal Resection] explode all trees | 80 |
| 6 | #4 OR #5 | 11132 |
| 7 | ("gastrectomy’’ OR ‘‘surgery" OR "resection surgery" OR "operation"):ti,ab,kw | 38247 |
| 8 | MeSH descriptor: [Gastrectomy] explode all trees | 1014 |
| 9 | #7 OR #8 | 39041 |
| 10 | MeSH descriptor: [Randomized Controlled Trial] explode all trees | 119 |
| 11 | MeSH descriptor: [Clinical Trial] explode all trees | 141 |
| 12 | MeSH descriptor: [Clinical Trials as Topic] explode all trees | 48156 |
| 13 | MeSH descriptor: [Double-Blind Method] explode all trees | 139772 |
| 14 | MeSH descriptor: [Single-Blind Method] explode all trees | 21312 |
| 15 | MeSH descriptor: [Multicenter Study] explode all trees | 10 |
| 16 | MeSH descriptor: [Multicenter Studies as Topic] explode all trees | 1889 |
| 17 | MeSH descriptor: [Placebos] explode all trees | 24210 |
| 18 | MeSH descriptor: [Epidemiologic Studies] explode all trees | 156304 |
| 19 | MeSH descriptor: [Observational Study] explode all trees | 3 |
| 20 | MeSH descriptor: [Observational Studies as Topic] explode all trees | 54 |
| 21 | MeSH descriptor: [Retrospective Studies] explode all trees | 8275 |
| 22 | Randomized OR Randomised OR Randomization OR Randomisation OR Random Allocation OR "Double Blind Method" OR "Single Blind Method" OR clinical trial OR multicenter study OR placebo OR randomly allocated OR Observational Study OR Observational OR Retrospective studies OR Epidemiologic Studies | 1368563 |
| 23 | #10 OR #11 OR #12 OR #13 OR #14 OR #15 OR #16 OR #17 OR #18 OR #19 OR #20 OR #21 OR #22 | 1368687 |
| 24 | #3 AND #6 AND #9 AND #23 | 47 |

# **Supplementary Table 2** Characteristics of studies excluded in the full-text review.

| Author, year | Ref | Country | Publication type | Study design | Inclusion | Patients, *n*  UD EGC/overall | | PSM | Long-term outcome | Reasons for exclusion |
| --- | --- | --- | --- | --- | --- | --- | --- | --- | --- | --- |
|  |  |  |  |  |  | ESD | Surgery |  |  |  |
| Chang, et al. 2017 | ([1](#_ENREF_1)) | Korea | Full-text article | Retrospective study | D EGC | 0/74 | 0/79 | No | OS, DSS, DFS, recurrence | Excluded UD EGC |
| Kim, et al. 2015 | ([2](#_ENREF_2)) | Korea | Full-text article | Retrospective study | D EGC | 0/165 | 0/292 | Yes | OS, DSS, recurrence | Excluded UD EGC |
| Pyo, et al. 2016 | ([3](#_ENREF_3)) | Korea | Full-text article | Retrospective study | D EGC | 0/1290 | 0/1273 | Yes | OS, DSS, DFS, recurrence | Excluded UD EGC |
| Ryu, et al. 2016 | ([4](#_ENREF_4)) | Korea | Full-text article | Retrospective study | D EGC | 0/81 | 0/144 | No | OS, DFS, recurrence | Excluded UD EGC |
| Shin, et al. 2017 | ([5](#_ENREF_5)) | Korea | Full-text article | Retrospective study | D EGC | 0/232 | 0/150 | Yes | OS, recurrence | Excluded UD EGC |
| Shim, et al. 2017 | ([6](#_ENREF_6)) | Korea | Conference abstract | Retrospective study | D EGC | 0/74 | 0/79 | No | OS, DFS | Excluded UD EGC |
| Bausys, et al. 2019 | ([7](#_ENREF_7)) | Lituania | Full-text article | Retrospective study | EGC | 1/42 | 104/218 | Yes | OS, DFS, recurrence | Did not specify UD EGC as a group or subgroup |
| Cho, et al. 2016 | ([8](#_ENREF_8)) | Korea | Full-text article | Retrospective study | EGC | 8/288 | 69/173 | Yes | OS, DFS, recurrence | Did not specify UD EGC as a group or subgroup |
| Kerdsirichairat et al. 2019 | ([9](#_ENREF_9)) | US,  Canada | Conference abstract | Retrospective study | EGC | 0/38 | 12/37 | No | OS, DSS, DFS, recurrence | Did not specify UD EGC as a group or subgroup |
| Kim, et al. 2014 | ([10](#_ENREF_10)) | Korea | Full-text article | Retrospective study | EGC | 15/142 | 17/71 | No | OS, DFS, recurrence | Did not specify UD EGC as a group or subgroup |
| Song, et al. 2015 | ([11](#_ENREF_11)) | China | Full-text article | Retrospective study | EGC | 3/29 | 12/59 | No | recurrence | Did not specify UD EGC as a group or subgroup |
| Zhao, et al. 2019 | ([12](#_ENREF_12)) | China | Full-text article | Retrospective study | EGC | 24/58 | 50/136 | No | OS, TFS, recurrence | Did not specify UD EGC as a group or subgroup |
| Rong, et al. 2018 | ([13](#_ENREF_13)) | China | Full-text article | Retrospective study | EGC | NA/74 | NA/96 | No | OS, recurrence | No data on the inclusion of UD EGC |
| Chiu, et al. 2012 | ([14](#_ENREF_14)) | Hong Kong | Full-text article | Retrospective study | EGC or dysplasia | NA/4 | NA/40 | No | OS, recurrence | No data on the inclusion of UD EGC |
| Chiu, et al. 2017 | ([15](#_ENREF_15)) | Hong Kong | Conference abstract | Randomized controlled trial | EGC or high grade dysplasia | NA/18 | NA/18 | No | OS, recurrence | No data on the inclusion of UD EGC |
| Hong, et al. 2020 | ([16](#_ENREF_16)) | Taiwan | Full-text article | Retrospective study | EGC or  severe dysplasia | NA/36 | NA/101 | No | DFS, DSS, recurrence | No data on the inclusion of UD EGC |
| Libanio, et al. 2019 | ([17](#_ENREF_17)) | Portugal | Full-text article | Prospective study | EGC or dysplasia | NA/153 | NA/101 | No | Recurrence | No data on the inclusion of UD EGC |
| Najmeg, et al. 2016 | ([18](#_ENREF_18)) | Canada | Full-text article | Retrospective study | EGC or dysplasia | NA/30 | NA/37 | No | OS, DFS, recurrence | No data on the inclusion of UD EGC |
| Choe, et al. 2019 | ([19](#_ENREF_19)) | Korea | Conference abstract | Retrospective study | EGC | NA/66 | NA/344 | No | OS | No data on the inclusion of UD EGC |
| Jung, et al. 2017 | ([20](#_ENREF_20)) | Korea | Conference abstract | Retrospective study | EGC | NA/551 | NA/692 | No | OS, DSS, recurrence | No data on the inclusion of UD EGC |
| Yang, et al. 2020 | ([21](#_ENREF_21)) | China | Full-text article | SEER database analysis | EGC | NA/475 | NA/2778 | Yes | DSS | No data on the inclusion of UD EGC |
| Yamashina, et al. 2015 | ([22](#_ENREF_22)) | Japan | Full-text article | Retrospective study | EGC in remnant stomach | 3/42 | 3/13 | No | OS, DSS, recurrence | Previous gastrectomy |
| Nonaka, et al. 2011 | ([23](#_ENREF_23)) | Japan | Full-text article | Retrospective study | EGC | NA/2052 | NA/2785 | No | None | Did not report long-term outcomes |
| Shimayoshi, et al. 2016 | ([24](#_ENREF_24)) | Japan | Conference abstract | Retrospective study | EGC | NA/362 | NA/231 | No | None | Did not report long-term outcomes |
| Park, et al. 2014 | ([25](#_ENREF_25)) | Korea | Full-text article | Retrospective study | EGC | 15/307 | 43/200 | Yes | OS, DFS, recurrence | Duplicated data |
| Chung, et al. 2014 | ([26](#_ENREF_26)) | Korea | Full-text article | Retrospective study | UD EGC | 76/76 | 149/149 | No | Recurrence | Duplicated data |
| Hahn, et al. 2018 | ([27](#_ENREF_27)) | Korea | Full-text article | Retrospective study | EGC | 93/817 | 564/1206 | No | OS, DSS, recurrence | Duplicated data |
| Kim, et al. 2018 | ([28](#_ENREF_28)) | Korea | Conference abstract | Retrospective study | UD EGC | 50/50 | 284/284 | No | Recurrence | Duplicated data |
| Jeon, et al. 2018 | ([29](#_ENREF_29)) | Korea | Full-text article | Retrospective study | EGC | 4/342 | 80/275 | Yes | OS, DSS, DFS, recurrence | Duplicated data |

*PSM*, propensity score matching; *ESD*, endoscopic submucosal dissection; *D EGC*, differentiated-type early gastric cancer; *OS*, overall survival; *DSS*, disease-specific survival; *DFS*, disease-free survival; *UD EGC*, undifferentiated-type early gastric cancer; *EGC*, early gastric cancer; *TFS,* treatment-free survival.

# **Supplementary Table 3** Sensitivity analysis using hazard ratio as a measure of outcome

| Outcome | Cohort | HR | 95% CI | I^2^ statistics |
| --- | --- | --- | --- | --- |
| OS | Overall | 2.07 | 1.04 – 4.11 | 0% |
| OS | PSM | 1.15 | 0.54 – 2.44 | 0% |
| DFS | Overall | 3.08 | 1.63 – 5.82 | 0% |
| DFS | PSM | 2.61 | 1.28 – 5.30 | 0% |
| Recurrence | Overall | 6.65 | 2.33 – 19.04 | 0% |
| Recurrence | PSM | 4.96 | 0.82 – 29.90 | 0% |
| Gastric recurrence | Overall | 4.80 | 1.56 – 14.74 | 0% |
| Gastric recurrence | PSM | 4.64 | 0.75 – 28.59 | 0% |
| Extragastric recurrence | Overall | 26.36 | 0.00 – 160226.34 | 0% |
| Extragastric recurrence | PSM | 68.37 | 0.00 – 6743628.54 | Not applicable |
| DSS | Overall | 4.01 | 1.32 – 12.13 | 0% |
| DSS | PSM | 61.01 | 0.00 – 5935067.44 | Not applicable |

*HR,* hazard ratio; *CI,* confidence interval; *OS*, overall survival; *DSS*, disease-specific survival; *DFS*, disease-free survival.

# **Supplementary Table 4** GRADE evidence table in the overall cohort

**Question:** ESD compared to Surgery for UD EGC

**Setting:** Comparison in overall cohort

| **Certainty assessment** | | | | | | | **№ of patients** | | **Effect** | | **Certainty** | **Importance** |
| --- | --- | --- | --- | --- | --- | --- | --- | --- | --- | --- | --- | --- |
| **№ of studies** | **Study design** | **Risk of bias** | **Inconsistency** | **Indirectness** | **Imprecision** | **Other considerations** | **ESD** | **Surgery** | **Relative (95% CI)** | **Absolute (95% CI)** |  |  |
| **Overall survival** | | | | | | | | | | | | |
| 5 | observational studies | very  serious^a^ | not serious | not serious | serious^b^ | none | 34/549 (6.2%) | 31/1314 (2.4%) | **RR 2.11** (1.26 to 3.55) | **0 fewer per 1,000** (from 0 fewer to 0 fewer) | ⨁◯◯◯ Very low | CRITICAL |
| **Disease-free survival** | | | | | | | | | | | | |
| 5 | observational studies | very  serious^a^ | not serious | not serious | serious^b^ | none | 61/549 (11.1%) | 38/1314 (2.9%) | **RR 3.27** (2.14 to 4.99) | **66 more per 1,000** (from 33 more to 115 more) | ⨁◯◯◯ Very low | CRITICAL |
| **Recurrence** | | | | | | | | | | | | |
| 5 | observational studies | very  serious^a^ | not serious | not serious | serious^b^ | none | 33/549 (6.0%) | 8/1314 (0.6%) | **RR 9.17** (4.02 to 20.92) | **50 more per 1,000** (from 18 more to 121 more) | ⨁◯◯◯ Very low | IMPORTANT |
| **Gastric recurrence** | | | | | | | | | | | | |
| 5 | observational studies | very  serious^a^ | not serious | not serious | serious^b^ | none | 28/549 (5.1%) | 7/1314 (0.5%) | **RR 8.39** (3.66 to 19.22) | **39 more per 1,000** (from 14 more to 97 more) | ⨁◯◯◯ Very low | IMPORTANT |
| **Extragastric recurrence** | | | | | | | | | | | | |
| 5 | observational studies | very  serious^a^ | not serious | not serious | serious^b^ | none | 5/549 (0.9%) | 1/1314 (0.1%) | **RR 5.15** (1.10 to 24.03) | **3 more per 1,000** (from 0 fewer to 18 more) | ⨁◯◯◯ Very low | CRITICAL |
| **Disease-specific survival** | | | | | | | | | | | | |
| 5 | observational studies | very  serious^a^ | not serious | not serious | serious^b^ | none | 4/549 (0.7%) | 2/1314 (0.2%) | **RR 4.21** (0.74 to 24.02) | **5 more per 1,000** (from 0 fewer to 35 more) | ⨁◯◯◯ Very low | CRITICAL |

**CI:** confidence interval; **RR:** risk ratio

**Explanations**

a. There were potential confounding factors that were significantly different between groups, but they were not controlled in the comparison of overall cohort.

b. The total number of events was less than 300.

# **Supplementary Table 5** GRADE evidence table in the propensity score matching cohort

**Question:** ESD compared to Surgery for UD EGC

**Setting:** Comparison in propensity score matching cohort

| **Certainty assessment** | | | | | | | **№ of patients** | | **Effect** | | **Certainty** | **Importance** |
| --- | --- | --- | --- | --- | --- | --- | --- | --- | --- | --- | --- | --- |
| **№ of studies** | **Study design** | **Risk of bias** | **Inconsistency** | **Indirectness** | **Imprecision** | **Other considerations** | **ESD** | **Surgery** | **Relative (95% CI)** | **Absolute (95% CI)** |  |  |
| **Overall survival** | | | | | | | | | | | | |
| 4 | observational studies | serious^a^ | not serious | not serious | serious^b^ | none | 17/400 (4.3%) | 16/508 (3.1%) | **RR 1.18** (0.60 to 2.32) | **6 more per 1,000** (from 13 fewer to 42 more) | ⨁⨁◯◯ Low | CRITICAL |
| **Disease-free survival** | | | | | | | | | | | | |
| 4 | observational studies | serious^a^ | not serious | not serious | serious^b^ | none | 38/400 (9.5%) | 17/508 (3.3%) | **RR 2.49** (1.42 to 4.35) | **50 more per 1,000** (from 14 more to 112 more) | ⨁⨁◯◯ Low | CRITICAL |
| **Recurrence** | | | | | | | | | | | | |
| 4 | observational studies | serious^a^ | not serious | not serious | serious^b^ | none | 25/400 (6.3%) | 1/508 (0.2%) | **RR 12.61** (3.43 to 46.37) | **23 more per 1,000** (from 5 more to 89 more) | ⨁⨁◯◯ Low | IMPORTANT |
| **Gastric recurrence** | | | | | | | | | | | | |
| 4 | observational studies | serious^a^ | not serious | not serious | serious^b^ | none | 22/400 (5.5%) | 1/508 (0.2%) | **RR 11.25** (3.06 to 41.40) | **20 more per 1,000** (from 4 more to 80 more) | ⨁⨁◯◯ Low | IMPORTANT |
| **Extragastric recurrence** | | | | | | | | | | | | |
| 4 | observational studies | serious^a^ | not serious | not serious | serious^b^ | none | 3/400 (0.8%) | 0/508 (0.0%) | **RR 4.23** (0.47 to 37.93) | **0 fewer per 1,000** (from 0 fewer to 0 fewer) | ⨁⨁◯◯ Low | CRITICAL |
| **Disease-specific survival** | | | | | | | | | | | | |
| 4 | observational studies | serious^a^ | not serious | not serious | serious^b^ | none | 3/400 (0.8%) | 0/508 (0.0%) | **RR 4.23** (0.47 to 37.93) | **0 fewer per 1,000** (from 0 fewer to 0 fewer) | ⨁⨁◯◯ Low | CRITICAL |

**CI:** confidence interval; **RR:** risk ratio

**Explanations**

a. Potential confounding factors that were significantly different between groups were controlled using propensity score matching analysis. However, there is still a risk of bias due to unmeasured potential confounding factors.

b. The total number of events was less than 300.

# **Supplementary Fig. 1** Risk of bias of studies included in the meta-analysis.


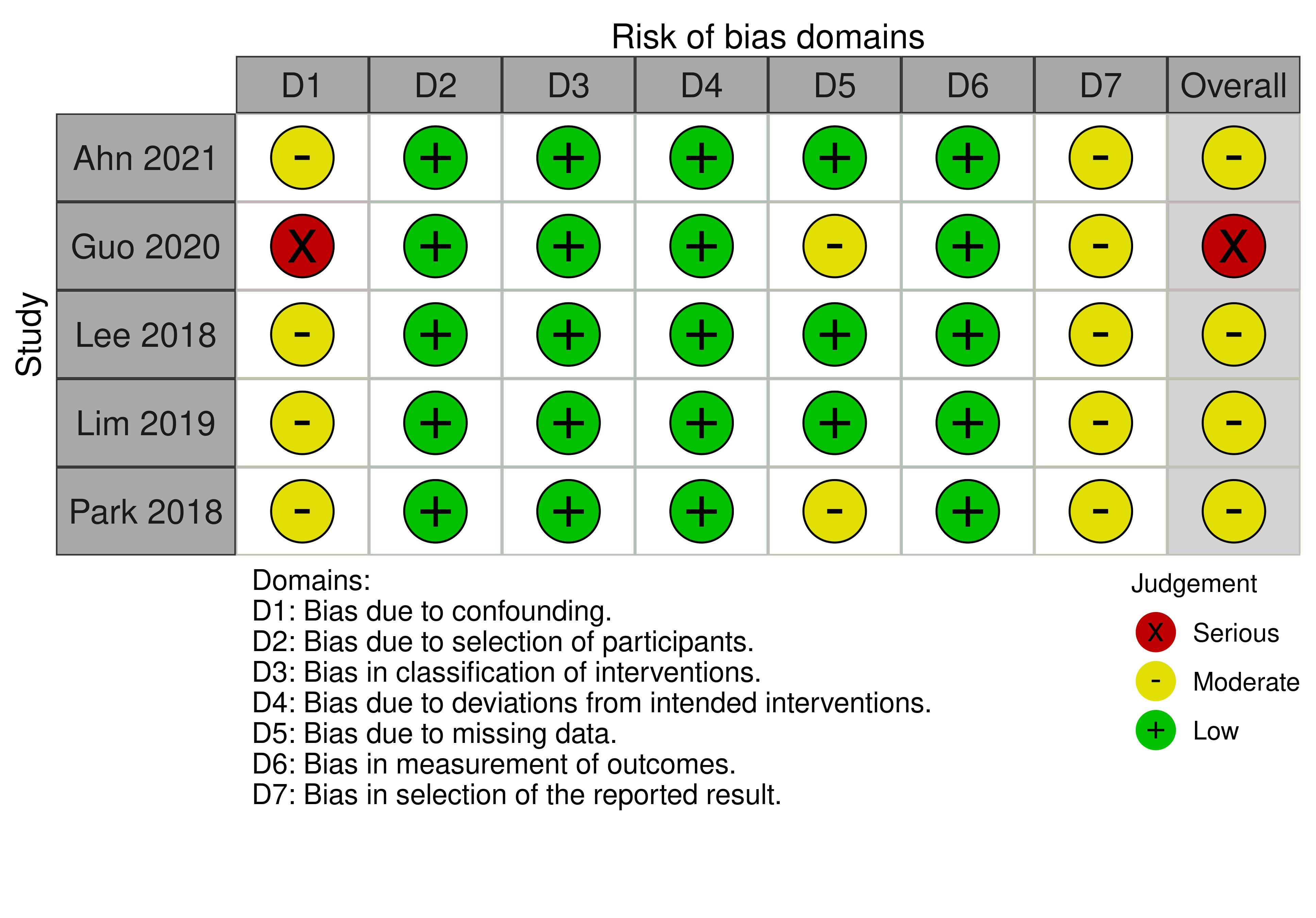


D1: Bias due to confounding. All known confounding domains including age, sex, comorbidities, and tumor-related factors were appropriately measured and controlled using propensity score matching analysis in Ahn 2021, Lee 2018, Lim 2019, and Park 2018. However, several important domains were not appropriately controlled in Guo 2020.

D2: Bias due to selection of participants. All participants eligible for target trial were included in the study, and follow-up was started on the date of intervention in all patients in all studies.

D3: Bias in classification of interventions. Intervention status was well defined as endoscopic submucosal dissection versus radical gastrectomy with lymph node dissection. This definition was based solely on information collected at the time of intervention.

D4: Bias due to deviations from intended interventions. There were not so many differences between the care provided to patients who underwent endoscopic submucosal dissection and patients who underwent surgical resection. Therefore, any deviations from the intended intervention reflected usual practice and were unlikely to impact the long-term outcomes including survival and recurrence.

D5: Bias due to missing data. Survival data were reasonably complete in Ahn 2021, Lee 2018, and Lim 2019 because survival status was obtained from national claims data as well as medical records. However, it was not in Guo 2020 and Park 2018, and the proportion of missing participants slightly higher in the endoscopic submucosal dissection group than in the surgery group in these studies.

D6: Bias in measurement of outcomes. The outcomes were survival and recurrence. The methods of outcome assessment were comparable between the groups and unlikely to be influenced by knowledge of the intervention received by study patients in all studies. In addition, any error in the outcome measurement is unrelated to intervention status because the outcome measures were objective.

D7: Bias in selection of the reported results. Because all studies were retrospective cohort studies, there was no pre-registered protocol or statistical analysis plan. However, the outcome measurements and analyses were clearly defined in the methods sections and consistent with those in the results sections in all studies. In addition, there were no indications of selection of reported outcomes among multiple analyses or selection of subgroups based on the results.

# **Supplementary Fig. 2** Subgroup analysis comparing poorly differentiated adenocarcinoma and signet ring cell carcinoma in overall survival between endoscopic submucosal dissection and surgery (**a**) in the overall cohort and (**b**) in the propensity score matching cohort.

ESD, endoscopic submucosal dissection.

(a)

(b)

# **Supplementary Fig. 3** Subgroup analysis comparing poorly differentiated adenocarcinoma and signet ring cell carcinoma in disease-free survival between endoscopic submucosal dissection and surgery (**a**) in the overall cohort and (**b**) in the propensity score matching cohort.

ESD, endoscopic submucosal dissection.

(a)

(b)

# **Supplementary Fig. 4** Subgroup analysis comparing poorly differentiated adenocarcinoma and signet ring cell carcinoma in (**a**) recurrence in the overall cohort, (**b**), recurrence in the propensity score matching cohort, (**c**) gastric recurrence in the overall cohort, (**d**) gastric recurrence in the propensity score matching cohort, (**e**) extragastric recurrence in the overall cohort, and (**f**) extragastric recurrence in the propensity score matching cohort between endoscopic submucosal dissection and surgery.

ESD, endoscopic submucosal dissection.

(a)

(b)

(c)

(d)

(e)

(f)

# **Supplementary Fig. 5** Subgroup analysis comparing poorly differentiated adenocarcinoma and signet ring cell carcinoma in disease-specific survival between endoscopic submucosal dissection and surgery (**a**) in the overall cohort and (**b**) in the propensity score matching cohort.

ESD, endoscopic submucosal dissection.

(a)

(b)

# **Supplementary Fig. 6** Sensitivity analysis using hazard ratio as a measure of outcome in overall survival between endoscopic submucosal dissection and surgery (**a**) in the overall cohort and (**b**) in the propensity score matching cohort.

ESD, endoscopic submucosal dissection.

(a)

(b)

# **Supplementary Fig. 7** Sensitivity analysis using hazard ratio as a measure of outcome in disease-free survival between endoscopic submucosal dissection and surgery (**a**) in the overall cohort and (**b**) in the propensity score matching cohort.

ESD, endoscopic submucosal dissection.

(a)

(b)

# **Supplementary Fig. 8** Sensitivity analysis using hazard ratio as a measure of outcome in (**a**) recurrence in the overall cohort, (**b**), recurrence in the propensity score matching cohort, (**c**) gastric recurrence in the overall cohort, (**d**) gastric recurrence in the propensity score matching cohort, (**e**) extragastric recurrence in the overall cohort, and (**f**) extragastric recurrence in the propensity score matching cohort between endoscopic submucosal dissection and surgery.

ESD, endoscopic submucosal dissection.

(a)

(b)

(c)

(d)

(e)

(f)

# **Supplementary Fig. 9** Sensitivity analysis using hazard ratio as a measure of outcome in disease-specific survival between endoscopic submucosal dissection and surgery (**a**) in the overall cohort and (**b**) in the propensity score matching cohort.

ESD, endoscopic submucosal dissection.

(a)

(b)

# **Supplementary Fig. 10** Funnel plots for overall survival (**a**) in the overall cohort and (**b**) in the propensity score matching cohort.

(a)

(b)

# **Supplementary Fig. 11** Funnel plots for disease-free survival (**a**) in the overall cohort and (**b**) in the propensity score matching cohort.

(a)

(b)

# **Supplementary Fig. 12** Funnel plots for (**a**) recurrence in the overall cohort, (**b**), recurrence in the propensity score matching cohort, (**c**) gastric recurrence in the overall cohort, (**d**) gastric recurrence in the propensity score matching cohort, (**e**) extragastric recurrence in the overall cohort, and (**f**) extragastric recurrence in the propensity score matching cohort.

(a)

(b)

(c)

(d)

(e)

(f)

# **Supplementary Fig. 13** Funnel plots for disease-specific survival (**a**) in the overall cohort and (**b**) in the propensity score matching cohort.

(a)

(b)

# **References**

1. Chang JY, Shim KN, Tae CH, Lee KE, Lee J, Lee KH, et al. Comparison of clinical outcomes after endoscopic submucosal dissection and surgery in the treatment of early gastric cancer: A single-institute study. Medicine (United States). 2017;96(30).

2. Kim YI, Kim YW, Choi IJ, Kim CG, Lee JY, Cho SJ, et al. Long-term survival after endoscopic resection versus surgery in early gastric cancers. Endoscopy. 2015;47(4):293-301.

3. Pyo JH, Lee H, Min BH, Lee JH, Choi MG, Lee JH, et al. Long-Term Outcome of Endoscopic Resection vs. Surgery for Early Gastric Cancer: A Non-inferiority-Matched Cohort Study. Am J Gastroenterol. 2016;111(2):240-9.

4. Ryu SJ, Kim BW, Kim BG, Kim JH, Kim JS, Kim JI, et al. Endoscopic submucosal dissection versus surgical resection for early gastric cancer: a retrospective multicenter study on immediate and long-term outcome over 5 years. Surgical Endoscopy. 2016;30(12):5283-9.

5. Shin DW, Hwang HY, Jeon SW. Comparison of Endoscopic Submucosal Dissection and Surgery for Differentiated Type Early Gastric Cancer within the Expanded Criteria. Clin Endosc. 2017;50(2):170-8.

6. Shim K, Chang J, Tae C, Moon C, Kim S, Jung H, et al. Comparison of clinical outcomes after endoscopic submucosal dissection and surgery in the treatment of early gastric cancer: A single-institute study. Helicobacter. 2017;22:69-70.

7. Bausys R, Bausys A, Stanaitis J, Vysniauskaite I, Maneikis K, Bausys B, et al. Propensity score-matched comparison of short-term and long-term outcomes between endoscopic submucosal dissection and surgery for treatment of early gastric cancer in a Western setting. Surgical Endoscopy. 2019;33(10):3228-37.

8. Cho JH, Cha SW, Kim HG, Lee TH, Cho JY, Ko WJ, et al. Long-term outcomes of endoscopic submucosal dissection for early gastric cancer: a comparison study to surgery using propensity score-matched analysis. Surg Endosc. 2016;30(9):3762-73.

9. Kerdsirichairat T, Wang R, Aihara H, Draganov PV, Kumta NA, Tomizawa Y, et al. Outcomes of endoscopic submucosal dissection versus surgery in elderly gastric cancer meeting standard and expanded indications: a multicenter North American cohort. Gastrointestinal Endoscopy. 2019;89(6):AB350-AB1.

10. Kim DY, Hong SJ, Cho GS, Jeong GA, Kim HK, Han JP, et al. Long-term efficacy of endoscopic submucosal dissection compared with surgery for early gastric cancer: A retrospective cohort study. Gut and Liver. 2014;8(5):519-25.

11. Song WC, Qiao XL, Gao XZ. A comparison of endoscopic submucosal dissection (ESD) and radical surgery for early gastric cancer: A retrospective study. World Journal of Surgical Oncology. 2015;13(1).

12. Zhao Y, Deng Z, Li H, Wang Y, Zhang W, Xiao Y, et al. A comparative study on endoscopic submucosal dissection and laparoscopy-assisted radical gastrectomy in the treatment of early gastric carcinoma. Journal of BUON. 2019;24(6):2506-13.

13. Rong L, Cai Y, Nian W, Wang X, Liang J, He Y, et al. Efficacy comparison between surgical resection and endoscopic submucosal dissection of early gastric cancer in a domestic single center. Zhonghua wei chang wai ke za zhi = Chinese journal of gastrointestinal surgery. 2018;21(2):190-5.

14. Chiu PWY, Teoh AYB, To KF, Wong SKH, Liu SYW, Lam CCH, et al. Endoscopic submucosal dissection (ESD) compared with gastrectomy for treatment of early gastric neoplasia: A retrospective cohort study. Surgical Endoscopy. 2012;26(12):3584-91.

15. Chiu PWYW, Teoh AY, Ng EK, Wong VW, Yip HC, Wu JC, et al. Endoscopic submucosal dissection compared to laparoscopic gastrectomy for treatment of early gastric cancer and a prospective randomized trial. Gastrointestinal Endoscopy. 2017;85(5):AB70-AB1.

16. Hong TC, Liou JM, Yeh CC, Yen HH, Wu MS, Lai IR, et al. Endoscopic submucosal dissection comparing with surgical resection in patients with early gastric cancer – A single center experience in Taiwan. Journal of the Formosan Medical Association. 2020;119(12):1750-7.

17. Libânio D, Braga V, Ferraz S, Castro R, Lage J, Pita I, et al. Prospective comparative study of endoscopic submucosal dissection and gastrectomy for early neoplastic lesions including patients' perspectives. Endoscopy. 2019;51(1):30-9.

18. Najmeh S, Cools-Lartigue J, Mueller C, Ferri LE. Comparing Laparoscopic to Endoscopic Resections for Early Gastric Cancer in a High Volume North American Center. Journal of gastrointestinal surgery : official journal of the Society for Surgery of the Alimentary Tract. 2016;20(9):1547-53.

19. Choe AR, Jung HK, Lim J, Tae CH, Moon CM, Kim SE, et al. A Survival analysis in elderly patients with gastric cancer according to treatment. Gastrointestinal Endoscopy. 2019;89(6):AB504.

20. Jung HI, Kim SJ, So IT, Choi JH, Lee YJ, Lee HJ, et al. Comparison of endoscopic submucosal dissection and surgery for the treatment of early gastric cancer: Single-center long-term outcome study. United European Gastroenterology Journal. 2017;5(5):A595.

21. Yang J, Ren M, Lu G, Liu H, Liu C, Wang W, et al. Gastrectomy versus endoscopic resection for patients with early-stage gastric adenocarcinoma: A population-based propensity matching study. Journal of Clinical Gastroenterology. 2020;54(10):871-8.

22. Yamashina T, Uedo N, Dainaka K, Aoi K, Matsuura N, Ito T, et al. Long-term survival after endoscopic resection for early gastric cancer in the remnant stomach: Comparison with radical surgery. Annals of Gastroenterology. 2015;28(1):66-71.

23. Nonaka S, Oda I, Nakaya T, Kusano C, Suzuki H, Yoshinaga S, et al. Clinical impact of a strategy involving endoscopic submucosal dissection for early gastric cancer: Determining the optimal pathway. Gastric Cancer. 2011;14(1):56-62.

24. Shimayoshi A, Yamamoto S, Kozumi K, Kimura E, Yoshida E, Sano F, et al. Early gastric cancer with mixed histological components is high-risk for lymph node metastasis. United European Gastroenterology Journal. 2016;4(5):A324.

25. Park CH, Lee H, Kim DW, Chung H, Park JC, Shin SK, et al. Clinical safety of endoscopic submucosal dissection compared with surgery in elderly patients with early gastric cancer: A propensity-matched analysis. Gastrointestinal Endoscopy. 2014;80(4):599-609.

26. Chung MW, Jeong O, Park YK, Lee KH, Lee JH, Lee WS, et al. [Comparison on the long term outcome between endoscopic submucosal dissection and surgical treatment for undifferentiated early gastric cancer]. The Korean journal of gastroenterology = Taehan Sohwagi Hakhoe chi. 2014;63(2):90-8.

27. Hahn KY, Park CH, Lee YK, Chung H, Park JC, Shin SK, et al. Comparative study between endoscopic submucosal dissection and surgery in patients with early gastric cancer. Surgical Endoscopy. 2018;32(1):73-86.

28. Kim J, Kim SG, Chung H, Jung HC. Comparison of endoscopic submucosal dissection and surgery for undifferentiatedtype early gastric cancer. Gastrointestinal Endoscopy. 2018;87(6):AB398.

29. Jeon HK, Kim GH, Lee BE, Park DY, Song GA, Kim DH, et al. Long-term outcome of endoscopic submucosal dissection is comparable to that of surgery for early gastric cancer: a propensity-matched analysis. Gastric Cancer. 2018;21(1):133-43.
